# Reduced structural connectivity in non-motor networks in children born preterm and the influence of early postnatal human cytomegalovirus infection

**DOI:** 10.3389/fneur.2023.1241387

**Published:** 2023-10-02

**Authors:** Pablo Pretzel, Marko Wilke, J-Donald Tournier, Rangmar Goelz, Karen Lidzba, Till-Karsten Hauser, Samuel Groeschel

**Affiliations:** ^1^Department of Child Neurology and Developmental Medicine, University Children’s Hospital, Tübingen, Germany; ^2^Experimental Pediatric Neuroimaging, Department of Child Neurology and Department of Neuroradiology, University Hospital, Tübingen, Germany; ^3^Centre for the Developing Brain, School of Biomedical Engineering and Imaging Sciences, King’s College London, London, United Kingdom; ^4^Biomedical Engineering Department, School of Biomedical Engineering and Imaging Sciences, King’s College London, London, United Kingdom; ^5^Department of Neonatology, University Children’s Hospital, Tübingen, Germany; ^6^Division of Neuropaediatrics, Development and Rehabilitation, Department of Paediatrics, Inselspital, Bern University Hospital, University of Bern, Bern, Switzerland; ^7^Department of Neuroradiology, University Hospital, Tübingen, Germany

**Keywords:** very early premature birth, postnatal cytomegalovirus infection, long-term effects, diffusion MRI, fixel-based analysis, tract-specific analysis, maternal transmission, pasteurization

## Abstract

**Introduction:**

Preterm birth is increasingly recognized to cause lifelong functional deficits, which often show no correlate in conventional MRI. In addition, early postnatal infection with human cytomegalovirus (hCMV) is being discussed as a possible cause for further impairments. In the present work, we used fixel-based analysis of diffusion-weighted MRI to assess long-term white matter alterations associated with preterm birth and/or early postnatal hCMV infection.

**Materials and methods:**

36 former preterms (PT, median age 14.8 years, median gestational age 28 weeks) and 18 healthy term-born controls (HC, median age 11.1 years) underwent high angular resolution DWI scans (1.5 T, *b *= 2 000 s/mm^2^, 60 directions) as well as clinical assessment. All subjects showed normal conventional MRI and normal motor function. Early postnatal hCMV infection status (CMV+ and CMV−) had been determined from repeated screening, ruling out congenital infections. Whole-brain analysis was performed, yielding fixel-wise metrics for fiber density (FD), fiber cross-section (FC), and fiber density and cross-section (FDC). Group differences were identified in a whole-brain analysis, followed by an analysis of tract-averaged metrics within *a priori* selected tracts associated with cognitive function. Both analyses were repeated while differentiating for postnatal hCMV infection status.

**Results:**

PT showed significant reductions of fixel metrics bilaterally in the cingulum, the genu corporis callosum and forceps minor, the capsula externa, and cerebellar and pontine structures. After including intracranial volume as a covariate, reductions remained significant in the cingulum. The tract-specific investigation revealed further reductions bilaterally in the superior longitudinal fasciculus and the uncinate fasciculus. When differentiating for hCMV infection status, no significant differences were found between CMV+ and CMV−. However, comparing CMV+ against HC, fixel metric reductions were of higher magnitude and of larger spatial extent than in CMV− against HC.

**Conclusion:**

Preterm birth can lead to long-lasting alterations of WM micro- and macrostructure, not visible on conventional MRI. Alterations are located predominantly in WM structures associated with cognitive function, likely underlying the cognitive deficits observed in our cohort. These observed structural alterations were more pronounced in preterms who suffered from early postnatal hCMV infection, in line with previous studies suggesting an additive effect.

## Highlights

- Preterm birth can lead to long-lasting alterations of white matter micro- and macrostructure, not visible on conventional MRI.
- White matter structures associated with cognitive function are predominantly affected.
- These white matter alterations are more pronounced in preterms who suffered from early postnatal cytomegalovirus infection.

## Introduction

Premature birth is a global challenge, with an estimated worldwide prevalence of 10.6%. Very premature birth, defined as gestational age below 32 weeks, accounts for approximately 15% of those cases and is associated with significant morbidity and mortality (1). While survival rates have improved over the last decades and rates of overt neurological impairment (such as cerebral palsy) are declining (2), very preterm born children remain at high risk for long-term neurodevelopmental impairments (3, 4). These “high prevalence, low severity dysfunctions” comprise a wide range of motor and cognitive domains (5) and have been shown to persist throughout adolescence and until early adulthood (6). This suggests that former preterms do not fully catch up with their term-born peers at a later developmental stage, but rather continue to carry the burden of premature birth throughout their entire life.

Numerous imaging studies sought to identify and characterize *in vivo* the neuropathology underlying these impairments, which encompasses both white matter (WM) and gray matter (GM) in a complex amalgam of dysmaturational disturbances (7). Diffusion-weighted MRI (dMRI) has proven especially useful when investigating the developing WM in preterm born children, as it detects microstructural alterations beyond those visible in conventional MRI (8, 9). A range of modeling techniques have been developed, aiming to derive interpretable metrics from dMRI data (10). Among these, fixel based analysis (FBA) has been recently introduced, with the key feature of fiber specificity of its metrics in white matter regions of complex spatial fiber arrangements (i.e., crossing fibers). In FBA, for each *fi*ber population within a vo*xel* (termed *fixel*), three quantitative metrics are defined, broadly representing microscopic change in intra-axonal water volume (fiber density, FD), macroscopic change in cross-section (fiber cross-section, FC), as well as combined micro- and macrostructural changes (fiber density and cross-section, FDC). These metrics can then be analyzed on the level of individual fixels, rather than averaged across an entire voxel, therefore allowing for a more meaningful investigation of WM microstructure throughout the entire brain. These aspects present a major improvement over approaches such as the commonly used diffusion tensor model (11), which suffers from limited interpretability in the presence of complex fiber arrangements due to the voxel-averaged nature of its metrics (10, 12). FBA was implemented in the MRtrix software (13), and has since been used in an increasing number of studies (10). A comprehensive discussion of the technique underlying FBA, as well as a thorough explanation and interpretation of its associated metrics, can be found in a recent review of FBA (10).

While preterm birth can impair brain development and lead to cognitive deficits, it can also leave affected children vulnerable to additional external influences in the immediate postnatal period. Among these, early postnatal infection with human cytomegalovirus (hCMV) is being discussed as a possible cause of further cognitive disabilities (14). While the severe predominantly neurologic complications of congenital hCMV infection are well known (15), as well as the mostly mild or asymptomatic course of postnatal infection in healthy, term infants given the transmission of maternal antibodies during the third trimester (16), the situation in very preterm infants is less clear (17). Neurodevelopmental comparisons of these with and without early postnatal hCMV infection did not show significant differences during infancy and young childhood, neither in cognitive nor in motor development (18, 19). However, long-term follow up investigations of that cohort revealed significantly reduced cognitive function in hCMV positive former preterms as measured by general IQ in adolescents (20), as well as a significant difference in motor function, albeit still within the normal range (21). Functional MRI studies also revealed differences between these groups (22). Taken together, these results indicate an additional adverse effect of early postnatal hCMV infection in very preterm born children. This warrants further investigation, especially given that transmission via breast milk can effectively be prevented in neonatal care units (23, 24).

In the present study, we aimed to investigate the long-term effects of very preterm birth on WM microstructure in this previously described cohort (20), as well as possible additional effects from early postnatal hCMV infection, using FBA. Our study cohort consists of children with mild cognitive deficits, but without gross motor deficits or overt pathology on conventional MRI. We therefore hypothesized that the microstructure of cognitive structural networks is altered as a consequence of preterm birth, and that early postnatal hCMV infection may be an aggravating factor.

## Materials and methods

### Participants

All patients (PT) were originally enrolled in a longitudinal cohort study on the long-term effects of early postnatal cytomegalovirus infection in preterm children (19–21, 25). PT were recruited from the neonatal intensive care unit in the University Children’s Hospital Tübingen, Germany, between 1995 and 2000. Inclusion criteria were very preterm birth (gestational age <32 weeks) or very low birth weight (<1,500 g).

For the present cross-sectional study, PT underwent follow-up clinical evaluation and MRI scans at school age. 18 term-born children between 7 and 17 years with typical development and normal neurological exam were recruited as healthy controls (HC). Exclusion criteria for both groups were psychiatric disorders, hearing deficits, and contraindications for MRI. HC were also excluded if their parents reported signs of infection or hepatopathy in the neonatal period, or if hearing impairment was present (all of which would indicate a possible congenital viral infection).

### Subject characterization

Cognitive abilities were assessed using the German version of the HAWIK-IV Wechsler Intelligence Scale for Children (26). This test yields a standardized full-scale IQ as well as 4 subscales (verbal comprehension, perceptual reasoning, processing speed and working memory).

The presence of Cerebral Palsy (CP) was determined clinically using the criteria of the Surveillance of Cerebral Palsy in Europe group (27).

Motor impairment was categorized using the Gross Motor Function Classification System (28), as well as with the Bimanual Fine Motor Function test (29).

Due to its known influence on functional outcome, maternal education level was assessed in years of education, as a proxy for socioeconomic status (30, 31). Handedness was assessed using the Edinburgh Handedness Inventory (32).

Congenital hCMV infection was ruled out in all PT via urine culture as well as ear and throat swabs immediately after birth. During their hospital stay, and again at 3 months corrected age, all PT were repeatedly screened for postnatal hCMV infection via urine culture and PCR tests. If these tests confirmed an early postnatal hCMV infection, PT were considered CMV+, otherwise CMV−.

Further details regarding subject enrollment and clinical assessments can be found in Brecht et al. (20) and Dorn et al. (22).

### Image acquisition and preprocessing

MRI data was acquired at the University Hospital of Tübingen, Germany, on a 1.5-T whole body MR scanner (Avanto, Siemens Medizintechnik, Erlangen, Germany), using a 12-channel head coil. Diffusion weighted imaging was obtained using a (twice-refocused) EPI sequence with TR = 6 900 ms, TE = 109 ms, with an isotropic set of 60 non-collinear directions, a diffusion-weighting factor of b = 2 000 s/mm^2^ and 45 contiguous axial slices with matrix = 80 × 80, resulting in a voxel size of 2.5 × 2.5 × 2.5 mm^3^. An experienced neuroradiologist (TKH) assessed conventional MRIs for overt lesions and signs of preterm brain injury. Direction-specific images were discarded if they exhibited motion artifacts (excessive blurring on visual inspection) in >5 slices. Total intracranial volume (TIV) was calculated using the automated volumetry provided by SPM (Wellcome Trust Centre for Neuroimaging, University College London, United Kingdom).

Following the recommended preprocessing for FBA studies (10), MRI images were denoised (33) and corrected for artifacts from Gibb’s ringing (34), subject motion and eddy currents (35, 36) and bias fields (37). Images were upsampled to an isotropic voxel size of 1.3 mm.

### Fixel-based metrics

Per-subject and cohort-averaged response functions were computed for gray and white matter as well as for cerebrospinal fluid (38). Based on these response functions, voxel-wise fiber orientation density (FOD) function maps were computed for each subject using multi-tissue constrained spherical deconvolution (39, 40), and intensity normalized to enable quantitative comparison of fixel metrics across all subjects.

A cohort-specific FOD template was then derived from all subject FOD maps using a nonlinear iterative registration and averaging approach (41, 42). From this FOD template, a cohort-specific fixel template for the subsequent fixel-based analysis was obtained by segmenting the individual FOD lobes (10).

Fixels and their associated FD values were extracted for each subject from their respective FOD maps in subject space. Subject FOD maps were then non-linearly registered into template space, and correspondence between subject and template fixels was determined. For each fixel, FC was calculated from the subject-to-template warp fields, and FDC was calculated as the product of its FD and FC values. All fixel values were smoothed based on fixel connectivity information (12) derived from a whole-brain tractogram of 2 million streamlines, which were generated using the probabilistic iFOD2 algorithm (43). All steps of this analysis pipeline followed recent recommendations (10).

### Tract-averaged metrics

As further explained below, we chose to investigate mean fixel metrics within a number of white matter tracts as part of our analysis. With regard to our hypothesis, we specifically selected WM tracts associated with cognitive functions: the arcuate and uncinate fasciculus, the cingulum, and the superior longitudinal fasciculus (SLF). Following the tripartite model of SLF architecture (44–46), we performed separate analyses for SLF I, II, and III. Similarly, we separated the arcuate fasciculus into a long, direct part, and a short, posterior, indirect part given the evidence of separate functional associations of these two parts (44).

To calculate tract-averaged fixel metrics, the whole-brain tractogram from the FBA pipeline was re-used to identify all streamlines within the WM tracts of interest. Inclusion and exclusion regions for streamline selection were chosen according to relevant literature (details are provided in Supplementary material), and identified using the SRI24 atlas (47), which was registered into template space. Spurious, anatomically implausible streamlines were removed. A 3D visualization of the resulting streamline selections can be found in Supplementary material. Average fixel metrics were then computed for all fixels within these selected streamlines.

### Statistical analysis

Initially, a whole-brain analysis was conducted, identifying the most affected structures throughout the entire WM without being limited to predefined regions of interest. To this end, regions of altered fixel metrics were identified using the framework of connectivity-based fixel enhancement (48). Fixel-wise statistical inference was calculated using a general linear model and non-parametric permutation testing over 5,000 permutations (49). Significance was assumed at p < 0.05, FWE-corrected for multiple comparisons.

First, all former preterms (PT) were compared to term healthy controls (HC), to identify WM alterations underlying prematurity *per se*. Then, to assess possible additional effects of early postnatal hCMV infection, additional comparisons were performed for CMV+ against CMV−, as well as separately for CMV+ and CMV− against HC. All analyses were repeated twice, once with age and sex as covariates, and once with total intracranial volume (TIV) as an additional nuisance variable, as suggested for FC and FDC (10).

MRtrix3 was used for image preprocessing, FBA, and visualization of FBA results (13).

The whole-brain approach, however, requires strict controlling for false positive results given the number of fixels. Therefore, in a second step, we analyzed tract-averaged fixel metrics within a number of white matter tracts selected at the outset of the study. At the expense of within-tract spatial resolution, this approach yields higher statistical sensitivity and can thus provide a more complete picture.

Tract-averaged fixel metrics were compared using an ANCOVA. Here, again, at first all PT were compared against HC, followed by individual comparisons of CMV+ against CMV− and separate comparisons of CMV+ and CMV− against HC. As in the FBA, all analyses were performed twice, once with sex and age as covariates, and in a second step with TIV as an additional covariate. Significance was assumed at p < 0.05, with Bonferroni correction applied for the number of investigated tracts.

R was used to perform the tract-averaged statistical analysis and to generate visualizations of the results (50).

## Results

44 patients were originally enrolled for prospective investigation (19). For the present, cross-sectional study, 36 children were available for follow-up examinations.

Demographic details of our study cohort are summarized in Table 1. All but two PT had a birth weight < 1,500 g, and all but one PT were born before the 32nd week. Notably, none had CP, and all PT presented with a GMFCS and BFMF of 0, indicating no overt motor dysfunction.

**Table 1:** Demographic details of all patients (PT) against healthy controls (HC).

	PT (*n* = 36)	HC (*n* = 18)	*p*
Males	25 (69%)	10 (56%)	0.314^(a)^
Age at assessment [years] (SD)	14.4 (1.4)	11.8 (3.1)	0.003^(b)^
EHI	0.8 (0.7–1)	0.8 (0.7–1)	0.763^(b)^
IQ (SD)	96.6 (17.9)	107.7 (8.3)	0.003^(c)^
MEL	12 (10–16)	18 (16–18)	0.001^(b)^
TIV [mL] (SD)	1417.2 (158.5)	1474.3 (145.2)	0.117^(b)^

Values are presented as mean (SD) or median (interquartile range). *p*-values are obtained from Pearson’s Chi-Square test ^(a)^, Wilcoxon rank-sum test ^(b)^, or Student’s *t*-test ^(c)^. EHI, Edinburgh Handedness Inventory; MEL, maternal education level; TIV, Total intracranial volume.

Distribution of subject sex and handedness did not differ between patients (PT) and healthy controls (HC). Significant differences were observed in maternal education level (MEL) and age. The difference in IQ has been discussed in a previous publication (20).

When differentiating for early postnatal hCMV infection (CMV+: 16 subjects, CMV−: 20 subjects), the groups were comparable, with no significant differences in any characteristic as summarized in Table 2.

**Table 2:** Demographic details of CMV+ against CMV− infants.

	CMV− (*n* = 20)	CMV+ (*n* = 16)	*p*
Males	14 (70%)	11 (69%)	0.936^(a)^
Age at assessment [years] (SD)	14.7 (1.1)	14.2 (1.6)	0.545^(b)^
EHI	0.8 (0.7–1)	0.8 (0.6–1)	0.843^(b)^
IQ	99 (20)	93.5 (15.1)	0.353^(c)^
MEL	12.5 (11.5–18)	12 (10–13.8)	0.517^(b)^
Birth weight [g] (SD)	963.7 (264.2)	1170.4 (355.4)	0.067^(b)^
Gestational Age [weeks] (SD)	27.6 (2.1)	28.4 (2.1)	0.238^(b)^
TIV [mL] (SD)	1426.4 (165.9)	1405.8 (153.3)	0.765^(b)^

Values are presented as mean (SD) or median (interquartile range). *p*-values are obtained from Pearson’s Chi-Square test ^(a)^, Wilcoxon rank-sum test ^(b)^, or Student’s *t*-test ^(c)^. EHI, Edinburgh Handedness Inventory; MEL, maternal education level; TIV, Total intracranial volume.

Visual inspection of conventional MRI scans showed normal white matter appearance and only minor sequelae of preterm birth, such as enlarged ventricles, in 7 PT subjects. The presence of gross pathology was ruled out in all subjects.

### Effect of prematurity

Comparing PT and HC in a whole-brain analysis with age and subject sex as covariates, we found significant differences in fixel-wise metrics. In Fiber Density (FD), we observed up to 30% relative reduction in both the right and left cingulum. Fiber cross-section (FC) was significantly reduced in the genu of the corpus callosum (CC) and forceps minor (up to 25% relative reduction), the left cingulum, the cerebellar-thalamic fibers (up to 15% relative reduction), the superior cerebellar peduncles (up to 10% relative reduction) and in the transverse pontine fibers (up to 10% relative reduction), as well as within the extreme capsule (up to 15% relative reduction). Fiber density and cross-section (FDC) was significantly reduced throughout both the right and left cingulum (up to 50% relative reduction), the genu corporis callosum and forceps minor (up to 40% reduction, with more extensive reductions in the left frontal lobe), and both the left and right external and extreme capsule (up to 40% reduction).

After incorporating total intracranial volume (TIV) as an additional covariate, alterations of FD and FDC remained significant in the left and right cingulum (relative reduction of up to 35% for FD, up to 60% for FDC), as well as within individual fixels of the external capsule (up to 25% relative reduction in FD). In FC, no alterations remained significant.

We did not find significantly increased FD, FC, or FDC in PT compared to HC.

**Figure 1:**
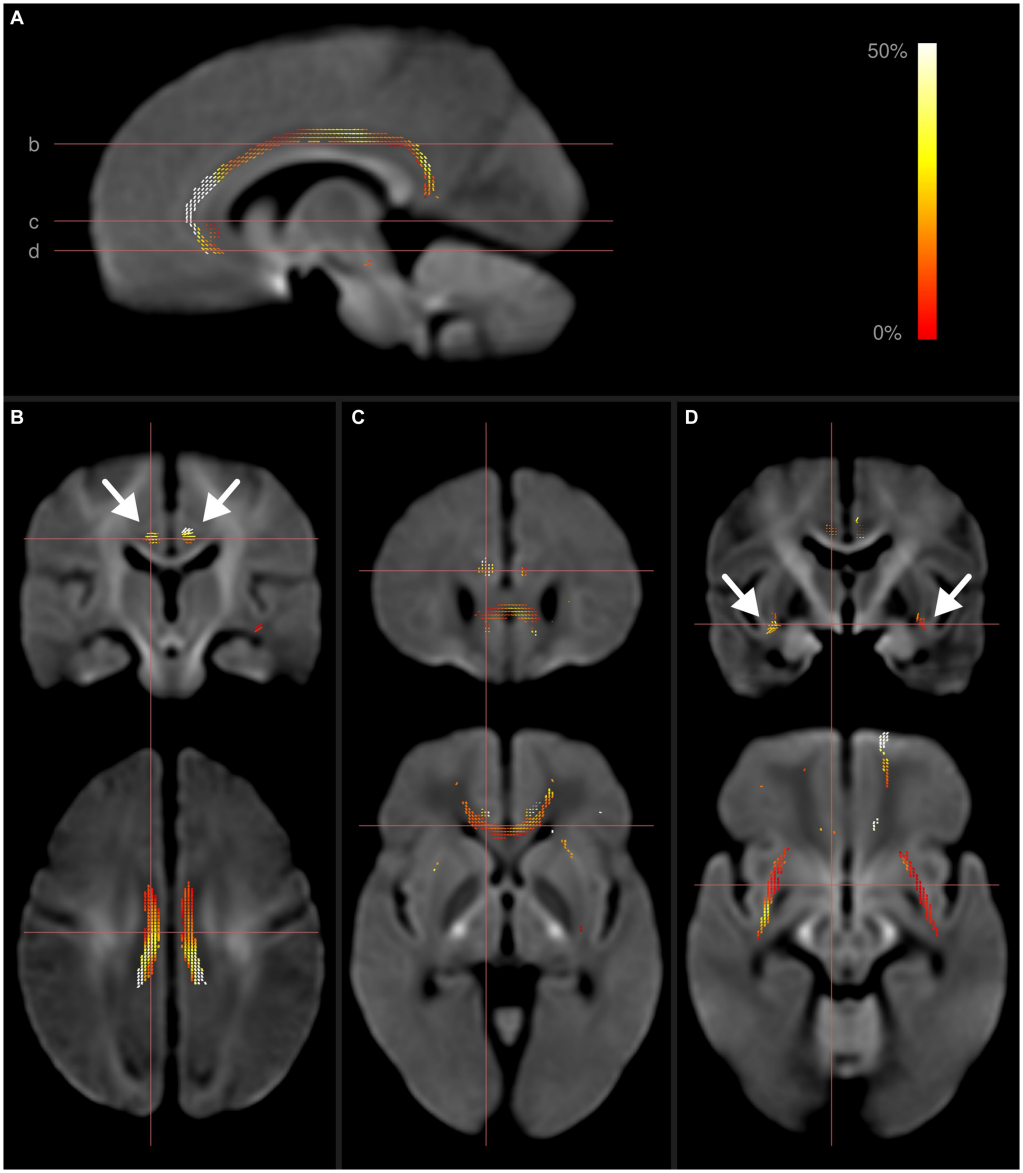
Fixel-wise whole-brain comparison of all preterms with term-born controls. Fixels with significantly reduced fiber density and cross-section (FDC) are colored by effect size, and overlaid on the cohort-specific template. The template contrast is derived from the template FODs. **(A)** Parasagittal view of FDC reductions in the cingulum. Horizontal lines show axial location of panels **(B–D)**. **(B)** Frontal and axial view of fixels with significantly reduced FDC in the cingulum. **(C)** Reduced FDC in the genu corporis callosum, forceps minor and cingulum. **(D)** Reduced FDC in the external capsule (arrows) and cingulum. Subject age and sex were included as covariates in this analysis. After including intracranial volume as an additional covariate, only the differences within the cingulum remained significant.

These results are illustrated in Figure 1. Additional figures detailing the individual whole-brain results for FD, FC, and FDC can be found in Supplementary material.

**Figure 2:**
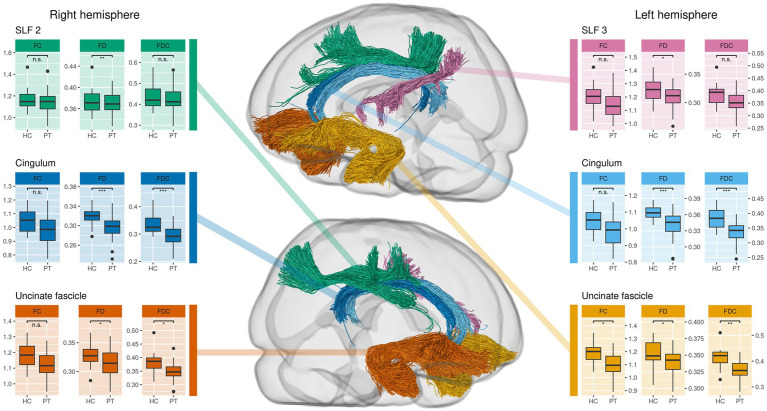
Comparison of tract-averaged fixel metrics between all preterms (PT) and healthy controls (HC). This approach complements the whole-brain analysis as it reveals additional alterations in uncinate and superior longitudinal fasciculus (SLF). Significance was determined using an ANCOVA with subject sex, age and total intracranial volume as covariates. Tracts not shown do not exhibit significant differences.

In the comparison of tract-averaged fixel metrics between PT and HC, we observed a significant reduction of FD bilaterally in the cingulum, in the right SLF2 and the left SLF3. FC was significantly reduced in the left uncinate fasciculus. For FDC, we found a bilateral significant reduction in the cingulate and uncinate fascicles. These findings are illustrated in Figure 2. Individual test results for each tract and metric can be found in Supplementary material.

### Effect of early postnatal hCMV infection

**Figure 3:**
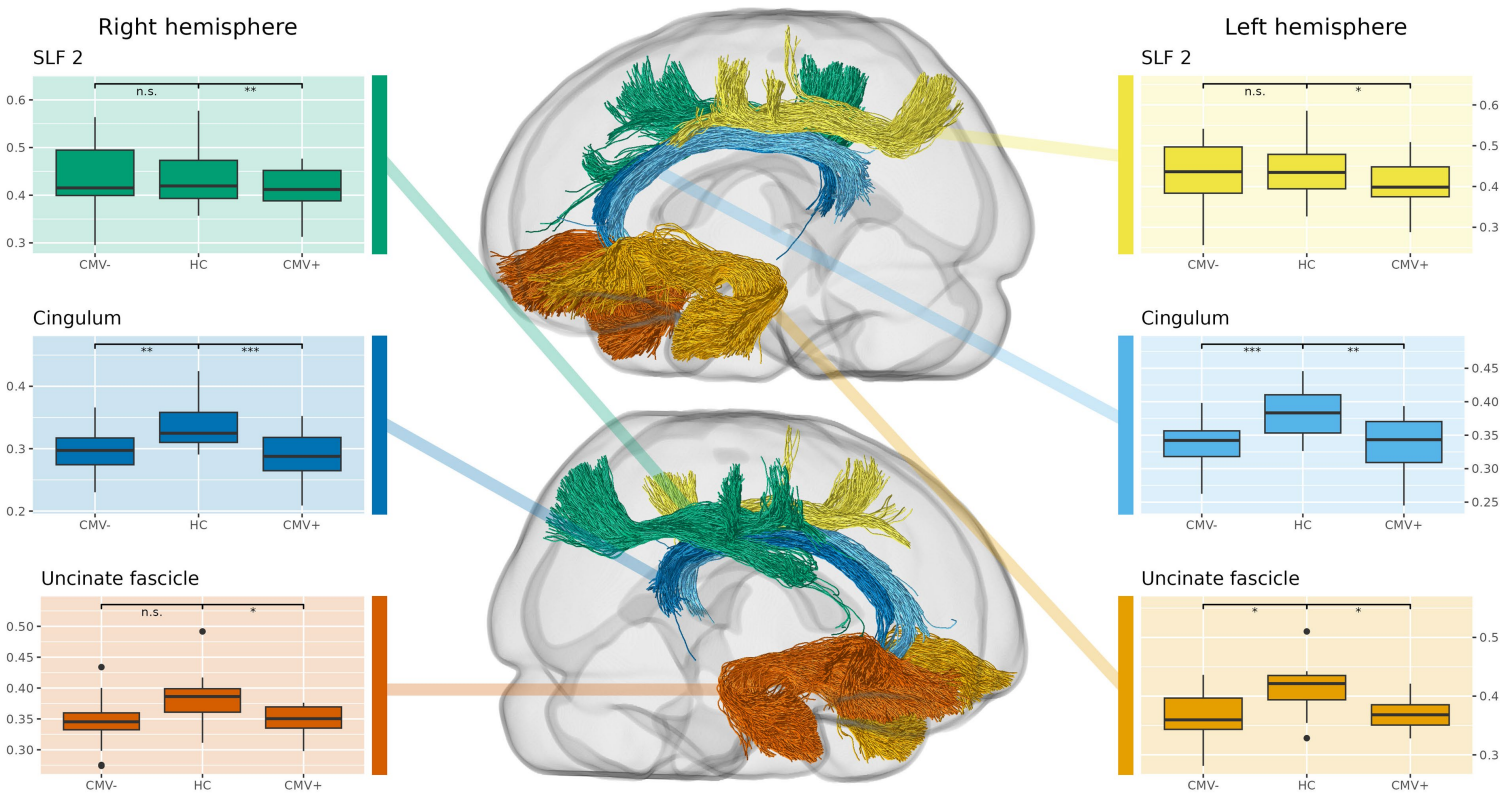
Comparison of tract-averaged fiber density and cross-section (FDC) in former preterms with (CMV+) and without (CMV−) early postnatal human cytomegalovirus infection, against term-born, healthy controls (HC). Again, this approach complements the whole-brain analysis, showing additional alterations in the uncinate and superior longitudinal fasciculus (SLF). Also, CMV+ show significantly reduced fixel metrics in a higher number of tracts than CMV−, suggesting that early postnatal hCMV infection leads to additional white matter alterations beyond those caused by prematurity alone. Tracts not shown do not express significant differences.

Directly comparing CMV+ against CMV−, the whole-brain analysis indicates reduced fixel metrics in CMV+ in the cerebellar peduncles and pontine fibers when lowering the significance threshold (see Supplementary Figures). The tract-averaged analysis further suggests reduced mean fixel metrics in CMV+ within SLF1 and SLF2 (see Figure 3). However, possibly due to the lack of statistical power given the small group sizes, none of these results reach significance, neither without nor after introducing TIV as an additional covariate.

The results of the separate comparisons of CMV+ and CMV− against HC largely coincided with the differences between all PT and HC, incidentally confirming the robustness of our findings even in these smaller groups. However, spatial extent and effect sizes of the WM alterations were generally larger in CMV+ than in CMV−.

When comparing CMV+ against HC, whole-brain analysis with sex and age as covariates showed significant reductions in FD in the cingulum (up to 40% relative reduction), in FC in the pons and the cerebellar peduncles (up to 15% relative reduction), and in FDC in the cingulum, the genu CC and the IFOF (each up to 40% relative reduction). After including TIV as a covariate, the differences in FC remained significant, while differences in FD and FDC did not.

The tract-averaged analysis of CMV+ against HC showed significant reductions of FD in both the left and right cingulum, as well as in the right SLF2. FC was significantly reduced in the right arcuate fasciculus and the left SLF2. Significant reductions in FDC were found in both the left and right cingulum and uncinate fasciculus, as well as in the right SLF2.

The whole-brain comparison of CMV− against HC showed significant differences in FC in the pons of up to 15%, and a reduction of up to 40% in FDC in the cingulum, the genu corpus callosum, and the IFOF. In all affected areas, differences were less extensive and relative reductions of fixel metrics were lower than in CMV+ against HC. No significant differences in FD were observed. When including TIV as a covariate, none of the differences in the WBA remained significant.

**Figure 4:**
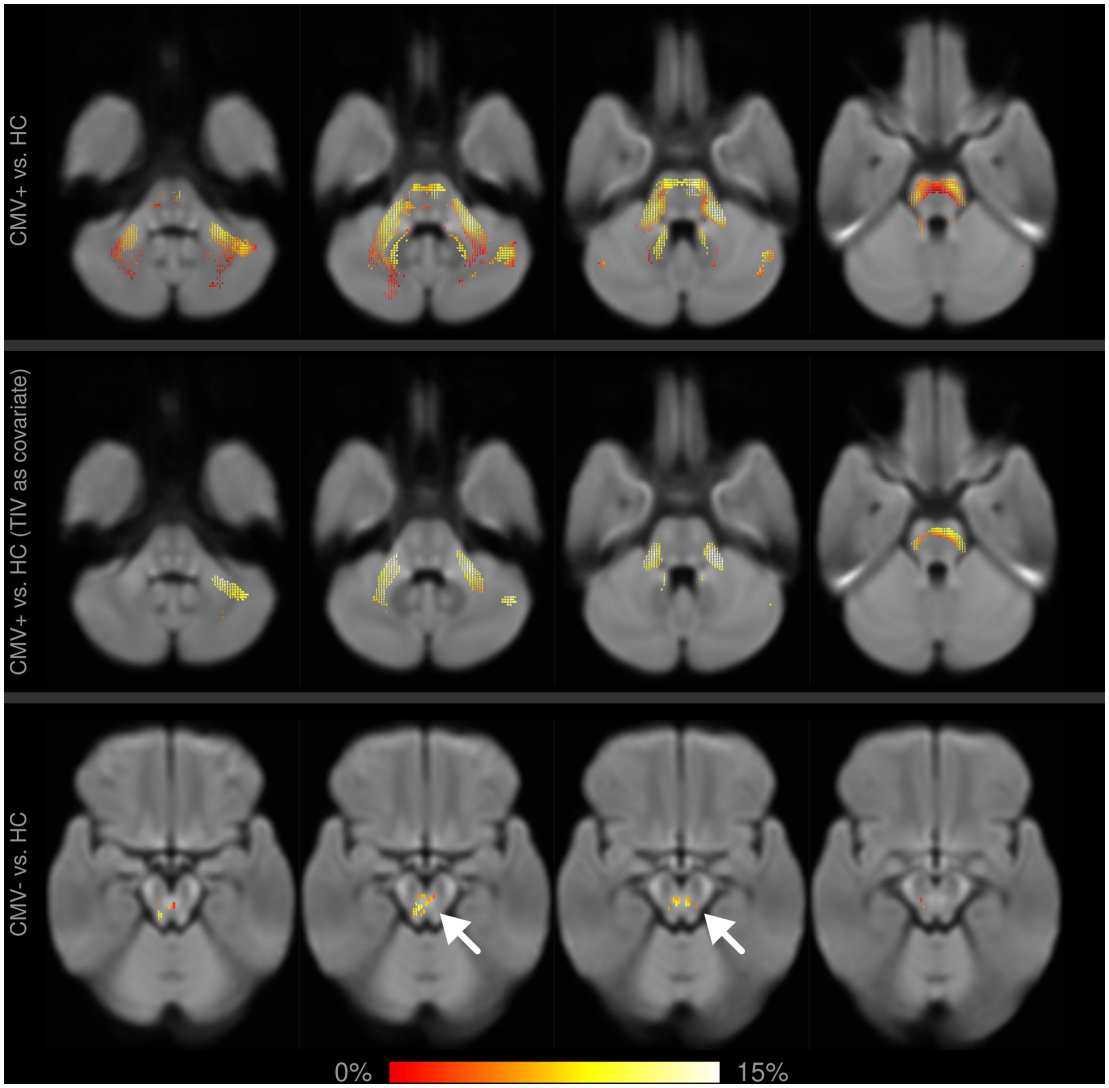
Fixel-wise whole-brain comparison of fiber cross-section (FC) between former preterms with (CMV+) and without (CMV−) early postnatal cytomegalovirus infection against healthy controls (HC). Fixels with significantly reduced FC are colored by effect size and overlaid on the cohort-specific template. Upper row: with age and sex as covariates, CMV+ show widespread alterations in the pons and cerebellar structures, which partly remain significant after including TIV as an additional covariate (middle row). Differences between CMV− and HC are much smaller in spatial extent (bottom row, arrows), and do not remain significant after adding TIV as a covariate.

In the tract-averaged comparison of fixel metrics between CMV− and HC, significant reductions were observed for FD in the left and right cingulum, the right SLF2 and the left SLF3. FDC was significantly reduced in both the left and right cingulum, as well as in the left uncinate fasciculus. Examples of the results in the whole-brain analysis are illustrated in Figure 4, the results of the comparison of tract-averaged metrics are illustrated in Figure 3. Individual results of these comparisons for all tracts and fixel metrics can be found in Supplementary material.

## Discussion

In this work on a well-defined cohort of school-aged children born very preterm, we show that premature birth can lead to long-term alterations in white matter tissue microstructure. Further, we present evidence for an additional detrimental effect of early postnatal hCMV infection. Using a hypothesis-free whole-brain analysis, we first identified the most severely affected structures. The subsequent tract-specific investigation, driven by our hypothesis of reduced structural connectivity within WM tracts of cognitive networks, then allowed for a closer, statistically more sensitive look at a set of WM structures selected *a priori* for their association with cognitive functions. With this combined approach, we were able to obtain a comprehensive picture encompassing all WM alterations in our study cohort, both as a function of prematurity and of early postnatal hCMV infection.

### Effect of prematurity

We detected distinct alterations in white matter tracts in our whole-brain approach (Figure 1). For all identified structures, associations with a diverse range of cognitive functions have been described (44, 51, 52). This is well in line with the clinical characteristics of our study cohort, which presented with a significant cognitive deficit in comparison to the term-born control group. Our results are also in line with previously described sequelae of preterm birth, as similar deficits in domains such as attention, working memory, or fluency, are a common observation in studies following the development of cognitive abilities in former preterm born children (53) and have been shown to persist well into adolescence and adulthood (6). Our results provide an indication of the WM pathology underlying these observations in our cohort, and potentially in preterm born children in general. This interpretation is strengthened further by the absence of fixel metric alterations within tracts associated with motor function, such as the corticospinal tract, which corresponds to the lack of motor impairments in our study group. Taken together, it seems plausible to conclude that the observed differences in micro- and macrostructural WM properties in our study cohort provide a possible (micro-)structural correlate of the differences in cognitive functions.

Alterations in fixel metrics were already observed at term-equivalent age in a study on preterm born children with little to no visual abnormalities on structural MRI (54). Here, after adjusting for TIV, differences were localized mainly throughout the corpus callosum, with only isolated differences in FC in other structures such as the cingulum or the cerebellum. In our cohort of school-aged children, we find differences in fixel metrics in a larger number of WM tracts. This could be explained by the different time points alone, but also by continuously impaired WM maturation after preterm birth leading to larger differences in fixel metrics later on, as maturation of WM tissue has been shown to correlate with changes in diffusion MRI metrics (55).

Further, the fact that only selective tracts show significant alterations might be explained by the unequal speed of maturation within different WM tracts (55). Especially the cingulum was shown to mature late, which is in line with our findings of the largest reductions within that structure. However, only longitudinal or several cross-sectional studies investigating the time course of WM development in former preterms would provide further insight into whether this is a temporary alteration (and former preterms catch up with term-born children at a later age), or whether that difference persists (or even increases) up until adulthood.

Our findings further complement a prior study of long-term alterations in fixel-based metrics (56). Notably, this study investigated WM fixel metrics in former preterms longitudinally at 7 and 13 years of age, providing further evidence for the above-mentioned impaired WM maturation after preterm birth. Their findings partially overlap with ours, but also encompass a number of additional WM tracts. In contrast to our study, however, the subjects from that cohort presented with a considerable number of abnormal findings in structural MRI scans. More overt anatomical abnormalities have previously been shown to correlate with larger differences in fixel metrics (54). The exclusion of such subjects in our study may therefore provide a “cleaner” picture, demonstrating that preterm birth leads to long-term alterations in micro- and macrostructural fixel metrics even in the absence of overt pathology in structural MRI.

The cingulum bundles were found to be altered in both hemispheres and all metrics, and consistently showed the largest effect sizes of all investigated structures. FD and FDC appeared to be primarily affected, while FC only exhibited a smaller effect size in the whole-brain analysis and did not present with significant differences in the tract-averaged analysis. This suggests loss of intra-axonal volume as the primary cause of these findings, while the cross-section of this bundle does not appear to be affected to the same degree by prematurity.

Given the nature of fixel metrics, these findings can be interpreted to reflect reduced capacity to relay information (12) within these tracts. As such, this finding nicely complements a previous, fMRI-based study on the same cohort (22). In that work, higher activation levels were observed in the anterior cingulate cortex and were interpreted as a higher cognitive load required to fulfill the study task. Impaired information transfer within the cingulum, which is comprised mostly of efferent connections from the cingulate cortex (52), could provide an explanation for these observations, as the higher activation in the cingulate cortex might be a compensatory reaction to counteract or overcome impaired information transfer along its WM connections. Both results point to alterations in the cingulum as a relevant contributor to cognitive impairments following prematurity, in line with previous studies (57).

The investigation of tract-averaged fixel metrics found alterations in further WM tracts associated with cognitive functions (Figure 2), highlighting the above-mentioned complementary relation of this method and the whole-brain analysis. These observations are particularly noteworthy in the case of the uncinate fasciculus, in which we found bilateral reductions in mean fixel metrics. Given its role in the language network (44), it provides further evidence for impaired structural connectivity in language networks (58) as a cause for language alterations in former preterms in general (59).

Differences in FC and FDC were also observed throughout further association fibers (Inferior fronto-occipital fasiculus and the capsula extrema), as well as within commissural fibers (forceps minor and genu of the corpus callosum) and infratentorial fiber bundles within the pons and cerebellum (Figure 1). However, after controlling for TIV as suggested (10), none of these differences remained statistically significant. This suggests that, while these structures might potentially be affected by preterm birth, the observed changes of fixel metrics were proportional to, and thus confounded by, the difference in TIV between the study groups. At the same time, the known association of premature birth with lower intracranial volume (60) introduces an implicit group encoding, leading to a potential underestimation of statistical significance. Our results therefore still serve as an indication of the long-term, widespread effects of premature birth on the WM throughout the developing brain.

As to the biological mechanisms underlying these results, prior literature suggests a “complex amalgam” of primary destructive and secondary dysmaturational disturbances as the result of preterm birth, specifically a combination of astrogliosis, microgliosis and hypomyelination, as well as axonal damage (7). Our findings are compatible with such processes—reductions in FD suggest a loss of intra-axonal volume (axonal damage), while mostly normal FC shows that the overall cross-section of the fiber bundles remained unchanged (gliosis replacing damaged axons). A similar conclusion was reached in a study investigating adults after premature birth (61). However, such conclusions must be drawn with caution, as studies correlating FBA metrics with histology are still rare, and, moreover, the MRI acquisition protocol does not allow a strict separation of FD and FC (10).

The significant difference in maternal education level (MEL) in our cohort (see Table 1) did not come unexpected, given the known association of MEL with both hCMV infection and preterm birth, and neurodevelopmental outcome (31). However, the association between MEL and brain morphometry is much weaker (62). As the focus of our work is mainly on the effect of prematurity and CMV on brain tissue, we did not specifically account for this group difference in our study.

### Effect of early postnatal hCMV infection

In this study, we further aimed to investigate whether early postnatal hCMV infection in very preterm born children has additional detrimental effects on long-term WM micro- and macrostructure, above and beyond those caused by prematurity alone. Here, the results were more subtle, as the direct comparison of fixel metrics between former preterms with and without early postnatal hCMV infection does not yield statistically significant differences, neither in the whole-brain analysis nor in the analysis of tract-averaged metrics.

However, after lowering the significance threshold, the whole-brain analysis suggests an additional negative impact of hCMV in the SLF and cerebellar structures (Supplementary Figures). Furthermore, in individual comparisons of these groups against HC, CMV+ appeared to be more affected than CMV−, with WM alterations being larger in spatial extent and expressing a higher relative reduction of fixel metrics in the whole-brain analysis (Figure 4). Similarly, in the tract-averaged analysis, a number of tracts only showed significant differences in CMV+ (Figure 3). Incidentally, these last two observations cannot be explained by differences in statistical power due to differences in group size—as CMV+ is the smaller group (*n* = 16 vs. *n* = 20 for CMV−), such effects would instead be expected in the opposite direction. Taken together, our results therefore qualitatively suggest that early postnatal hCMV infection after prematurity does indeed negatively affect WM micro- and macrostructure and leads to more pronounced long-term alterations. Formal statistical significance, though, cannot be proven.

These observations bear a resemblance to an earlier study on fMRI metrics in the same cohort (22). Here, also, only minimal differences between preterms with and without early postnatal hCMV infection were found, but differences were more pronounced in those with hCMV infection. Both that study as well as the present work potentially suffer from limited statistical power and sensitivity due to the rather small group sizes after differentiating for hCMV infection status.

A very recent study investigated the effect of postnatal hCMV infection on brain structure in 18 preterm-born children at term-equivalent age and found no significant differences (63). While comparable to our study in cohort size, this study used diffusion tensor imaging parameters with their known limitations regarding sensitivity of detecting more subtle microstructural abnormalities compared to our approach using fixel-based metrics (10). Furthermore, an investigation at term-equivalent age is unable to detect differences that might manifest only during brain development later in life.

The exact pathomechanism of white matter damage in early postnatal hCMV infection in preterms is unclear, although similarities to white matter damage resulting from prematurity itself have been suggested (64). Given that the timing of both insults is similar, this must be expected, but not enough data (such as perinatal and longitudinal imaging) is available from this cohort to support or refute this suggestion.

Taken together, even in the absence of statistically significant differences in the direct group comparison, the consistent direction of our findings (in all comparisons, CMV+ were more affected than CMV−) suggests that there is indeed a long-term influence of early postnatal hCMV infection on the developing white matter, which might serve as an indication of possible structural correlates for the observed 10-point IQ deficit in CMV+ in comparison to CMV− (20). Given that early postnatal hCMV infection can be avoided by simple preventative measures in at-risk newborns (65, 66), this question requires further attention. Future studies should attempt to investigate larger cohorts and to use MRI sequences specifically tailored to the needs of fixel-based analysis, with higher b-values and higher MRI field strength.

### Strengths and limitations

A specific strength of this study is the use of FBA as an advanced analysis method for diffusion MRI. It enables a fiber-specific characterization of structural WM alterations, especially in the presence of complex fiber arrangements (10), which would have pushed tensor-based approaches to the limits of interpretability.

Furthermore, our single-center study cohort is well defined and specifically suited to answer our research question, with slight cognitive deficits but no gross motor function deficits. Our cohort results from a rigorous screening regime to differentiate PT into CMV+ and CMV− and to rule out congenital hCMV infections (25).

The age difference between PT and HC, with PT being significantly older (see also Table 1), is a somewhat limiting factor with this study. While this could have been ameliorated at least in part by excluding the youngest subjects in the HC group, thus creating a more even age distribution, we opted not to do so to maximize statistical power. To account for the difference, however, age was included as a covariate in all group comparisons. At the same time, WM maturation is associated with rising fixel metrics (56), therefore, given the age difference, PT would be expected to present with higher fixel metrics on average. This consideration substantiates our findings of reduced fixel metrics, meaning that our results err on the conservative side, under-rather than overestimating group differences.

## Conclusion

Preterm birth can cause long-term cognitive deficits, in many cases without a visible correlate in conventional MRI. We showed that affected children exhibit white matter alterations in structures such as the cingulum, arcuate and uncinate fasciculus and the superior longitudinal fasciculus. All of these structures are associated with cognitive function, therefore suggesting that these findings underlie the observed cognitive deficits. Furthermore, as children with early postnatal cytomegalovirus infection exhibit more pronounced white matter alterations, an additional effect of such an infection seems plausible and preventative measures should be considered.

## Data availability statement

The datasets presented in this article are not readily available due to lack of explicit written permission from participants and their legal guardians. Requests to access the datasets should be directed to the corresponding author.

## Ethics statement

The studies involving humans were approved by Ethics Committee of the University of Tübingen, Germany (decision no. 216/2009BO). The studies were conducted in accordance with the local legislation and institutional requirements. Written informed consent for participation in this study was provided by the participants’ legal guardians/next of kin.

## Author contributions

PP, SG, MW, RG, and KL contributed to the conception and design of the study. SG, MW, KL, RG, and T-KH contributed to data acquisition. PP and SG performed analysis and interpretation, MW and J-DT contributed to analysis and interpretation of data. PP wrote the first draft of the manuscript. All authors contributed to the article and approved the submitted version.
