# Supplementary material for: Reduced structural connectivity in non-motor networks in children born preterm and the influence of early postnatal human cytomegalovirus infection
Source: Front Neurol. 2023 Oct 2;14:1241387. doi: 10.3389/fneur.2023.1241387 (PMC10577195; doi:10.3389/fneur.2023.1241387)
Supplement: Supplementary file 1 [file Data_Sheet_1.PDF]

Reduced structural connectivity in non-motor networks in children born preterm and the influence of early postnatal hCMV infection

Supplementary Figures: Whole-brain analysis results

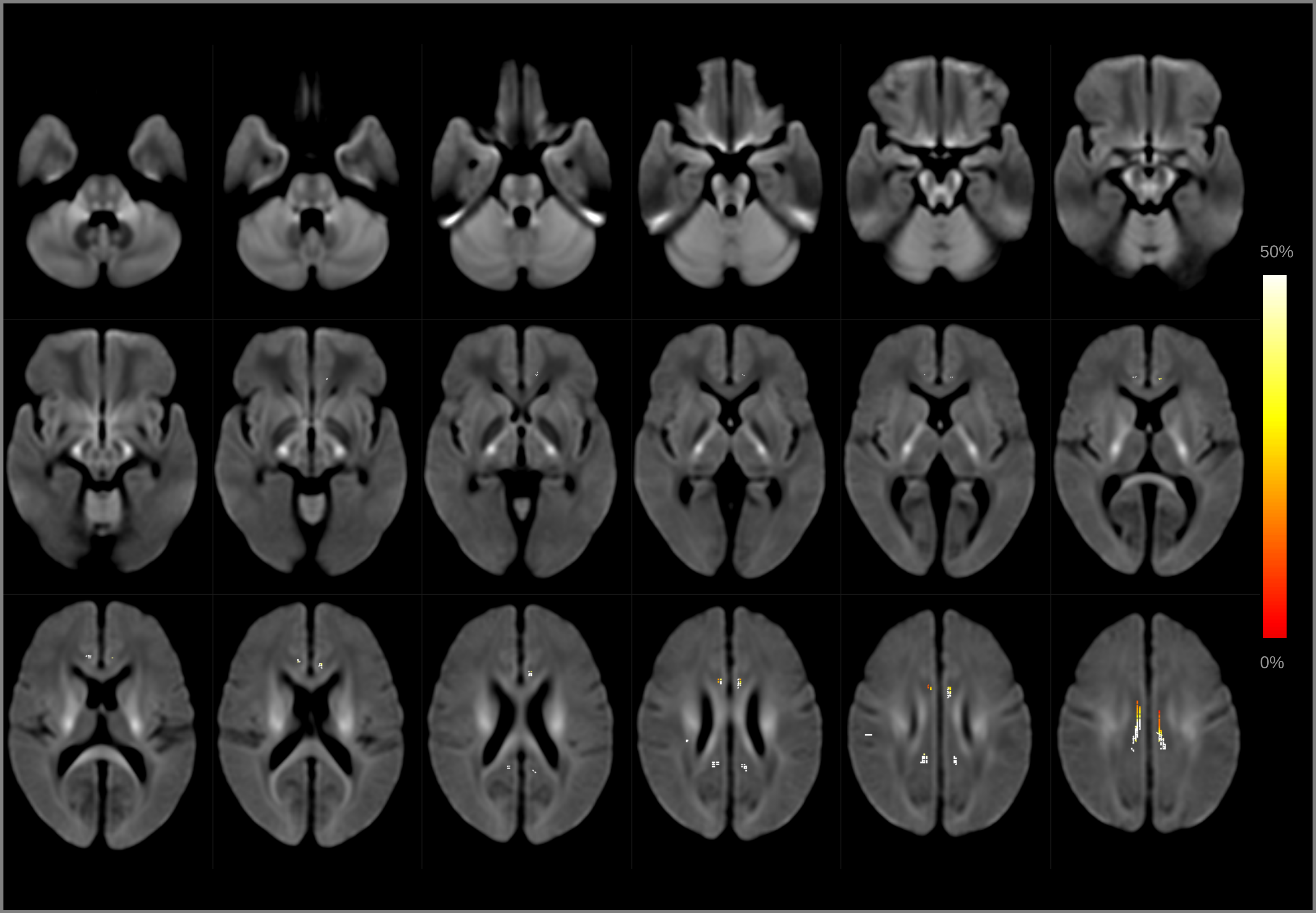

Figure 1: Whole-brain analysis of fixel-wise differences in FD (Fiber Density) between all preterms and controls, using a general linear model with age and sex as covariates.

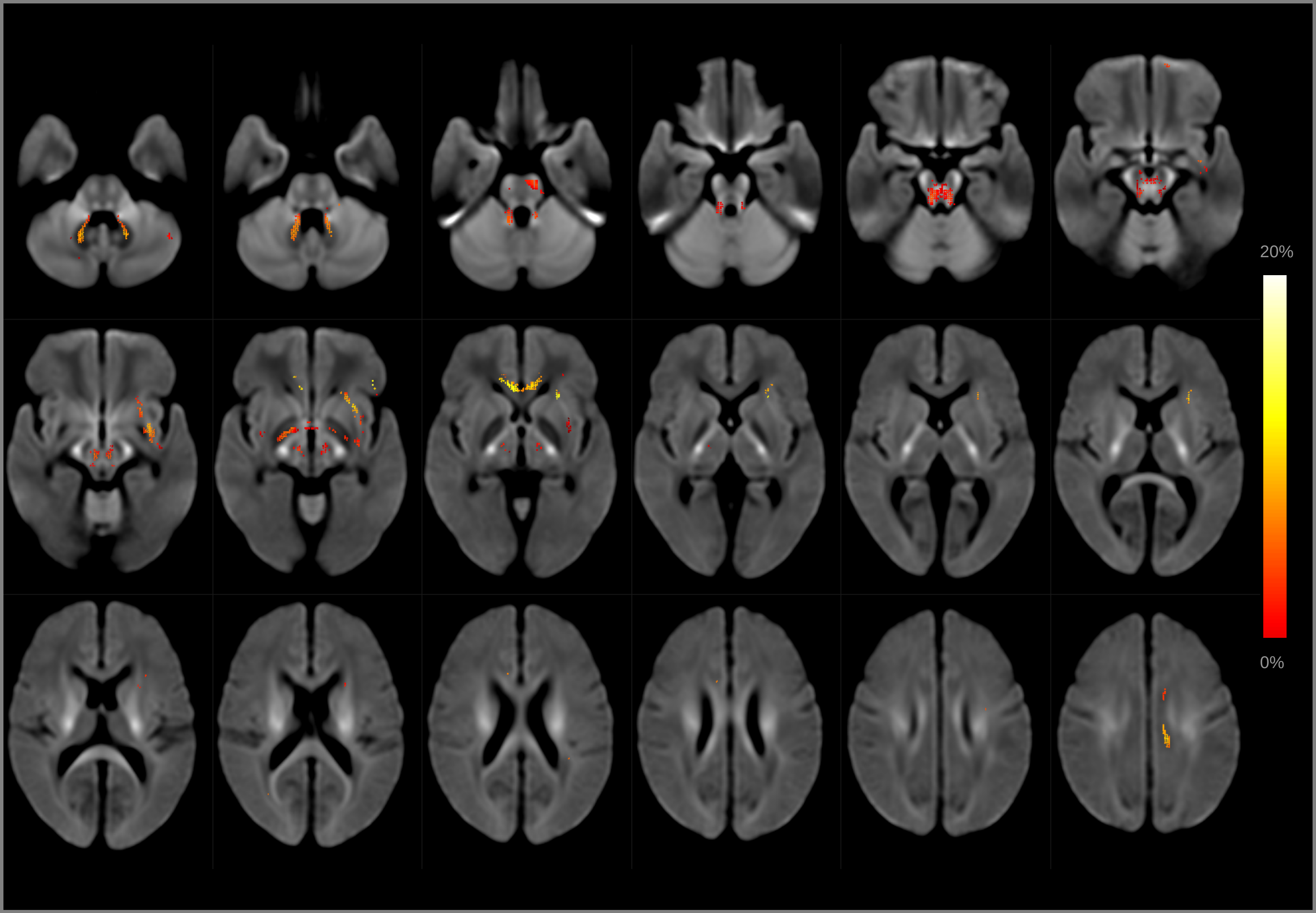

Figure 2: Whole-brain analysis of fixel-wise differences in FC (Fiber Cross-section) between all preterms and controls, using a general linear model with age and sex as covariates.

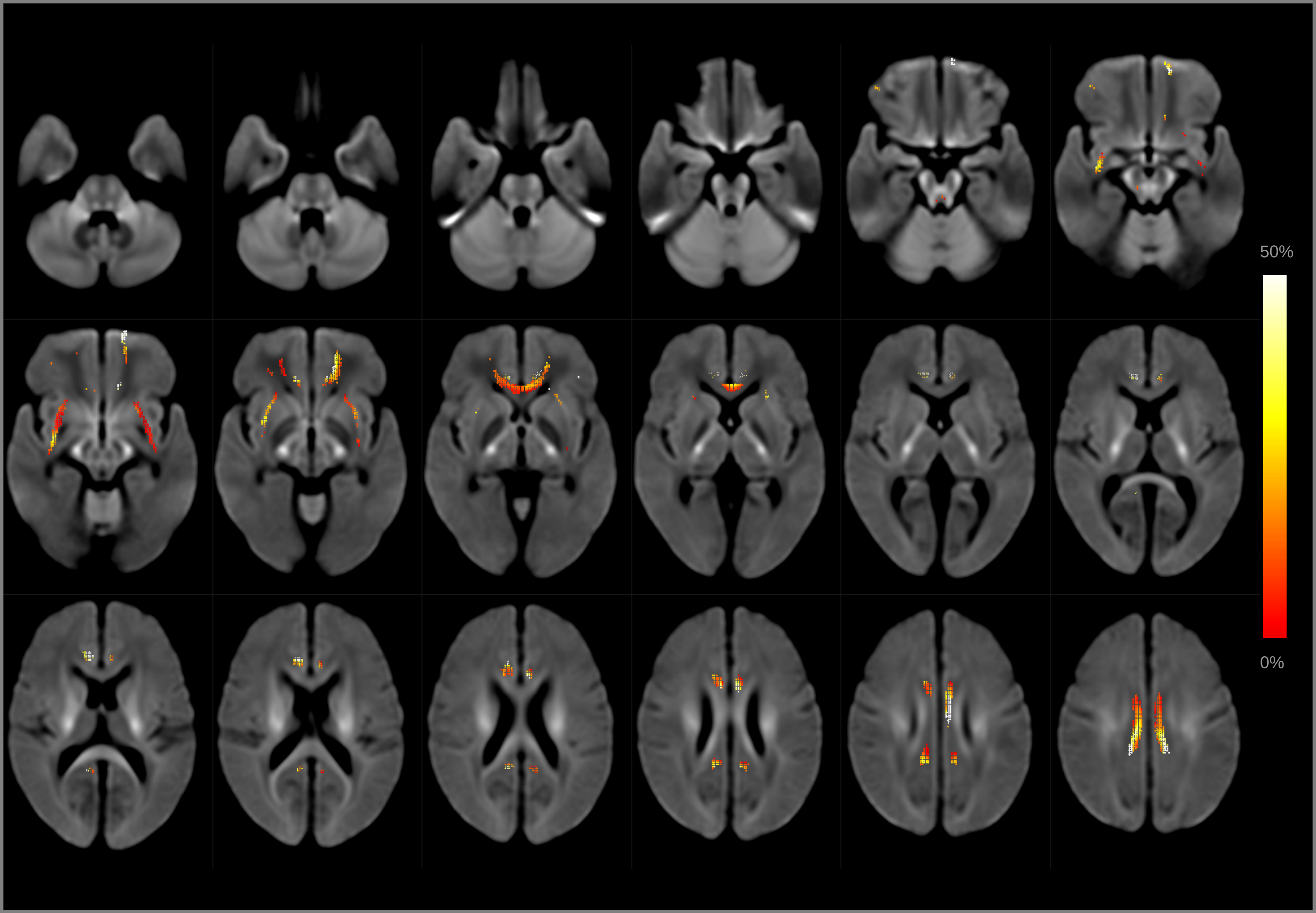

Figure 3: Whole-brain analysis of fixel-wise differences in FDC (Fiber Density and Cross-section) between all preterms and controls, using a general linear model with age and sex as covariates.

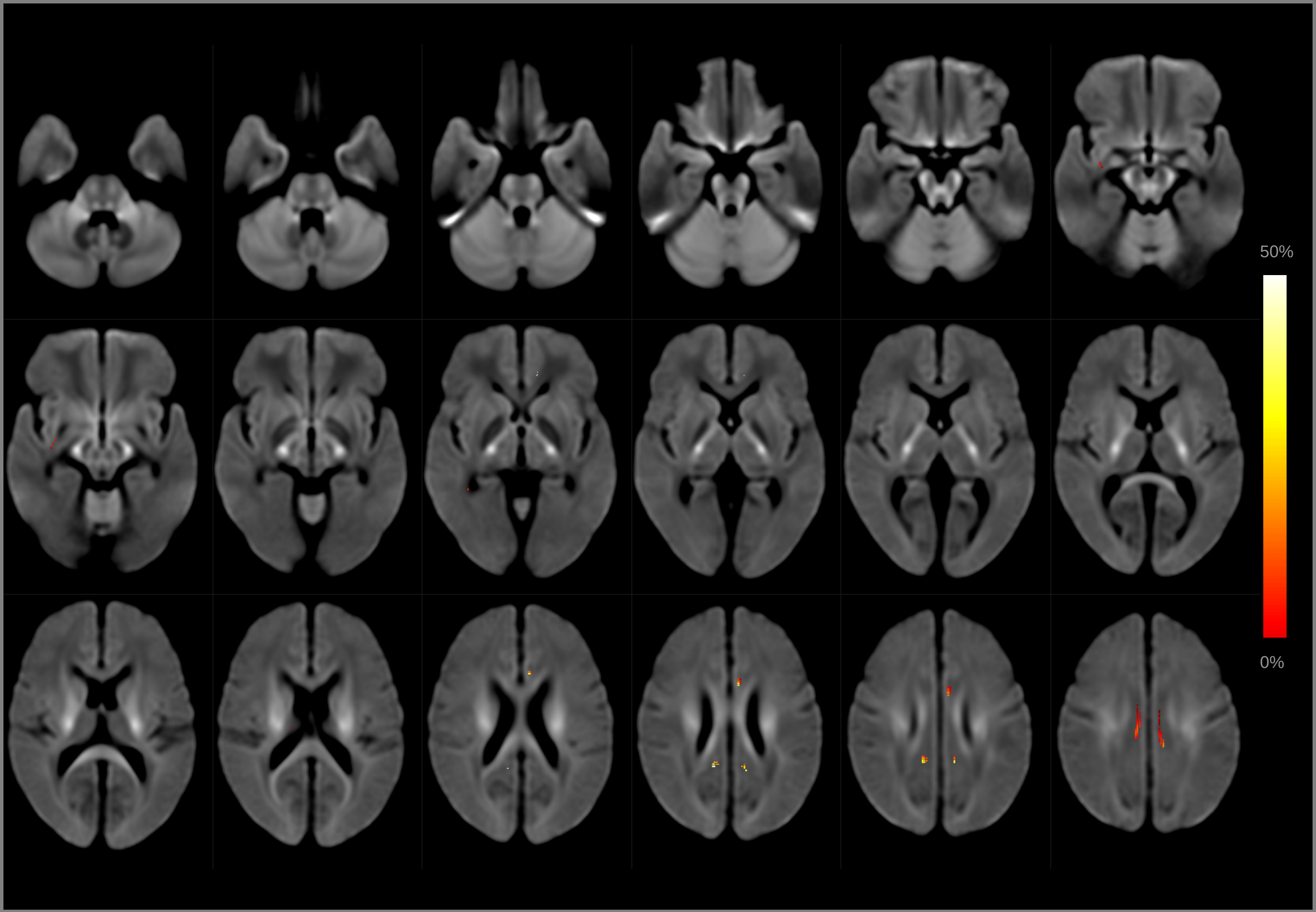

Figure 4: Whole-brain analysis of fixel-wise differences in FD (Fiber Density) between all preterms and controls, using a general linear model with age, sex and total intracranial volume (TIV) as covariates.

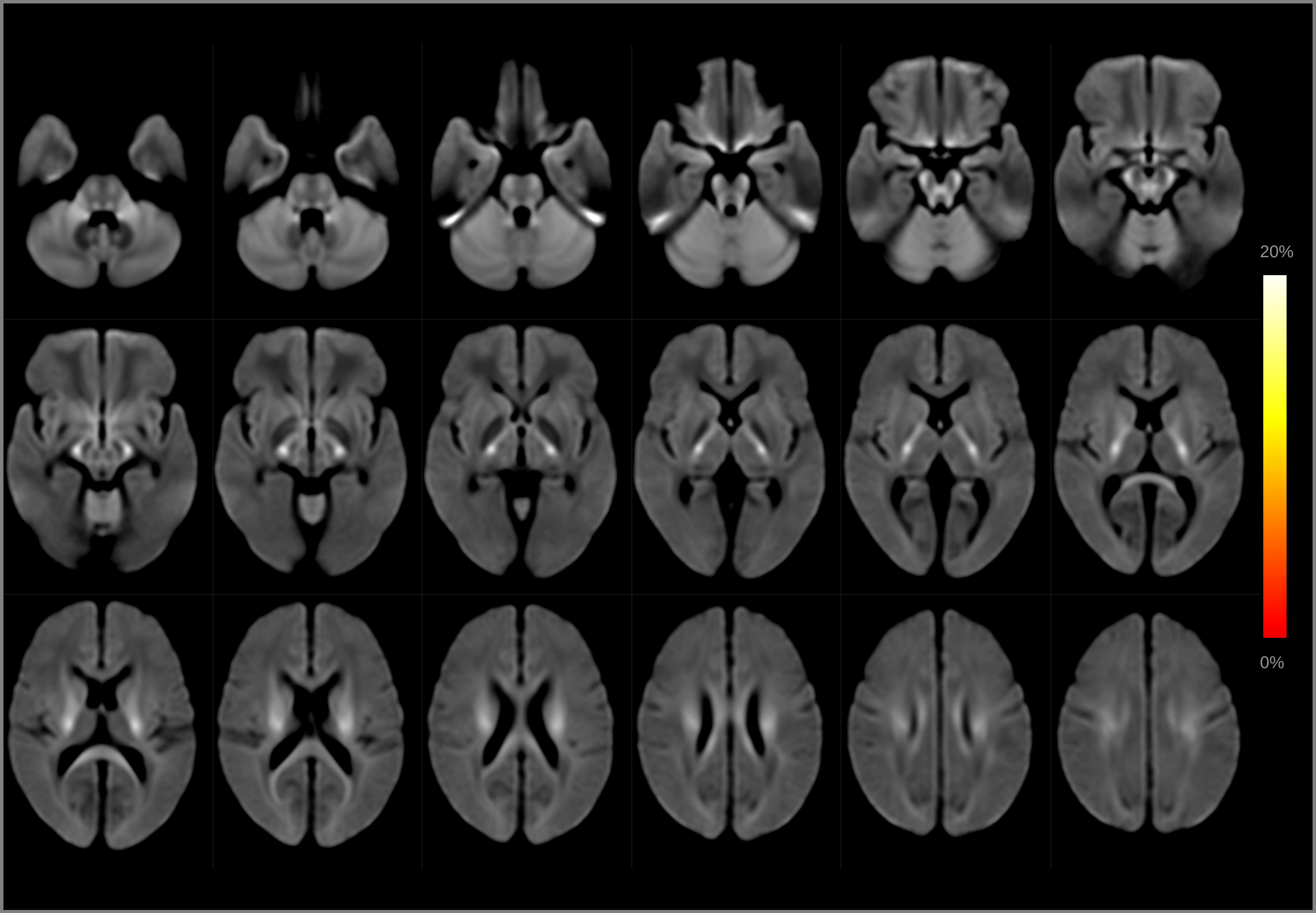

Figure 5: Whole-brain analysis of fixel-wise differences in FC (Fiber Cross-section) between all preterms and controls, using a general linear model with age, sex and total intracranial volume (TIV) as covariates.

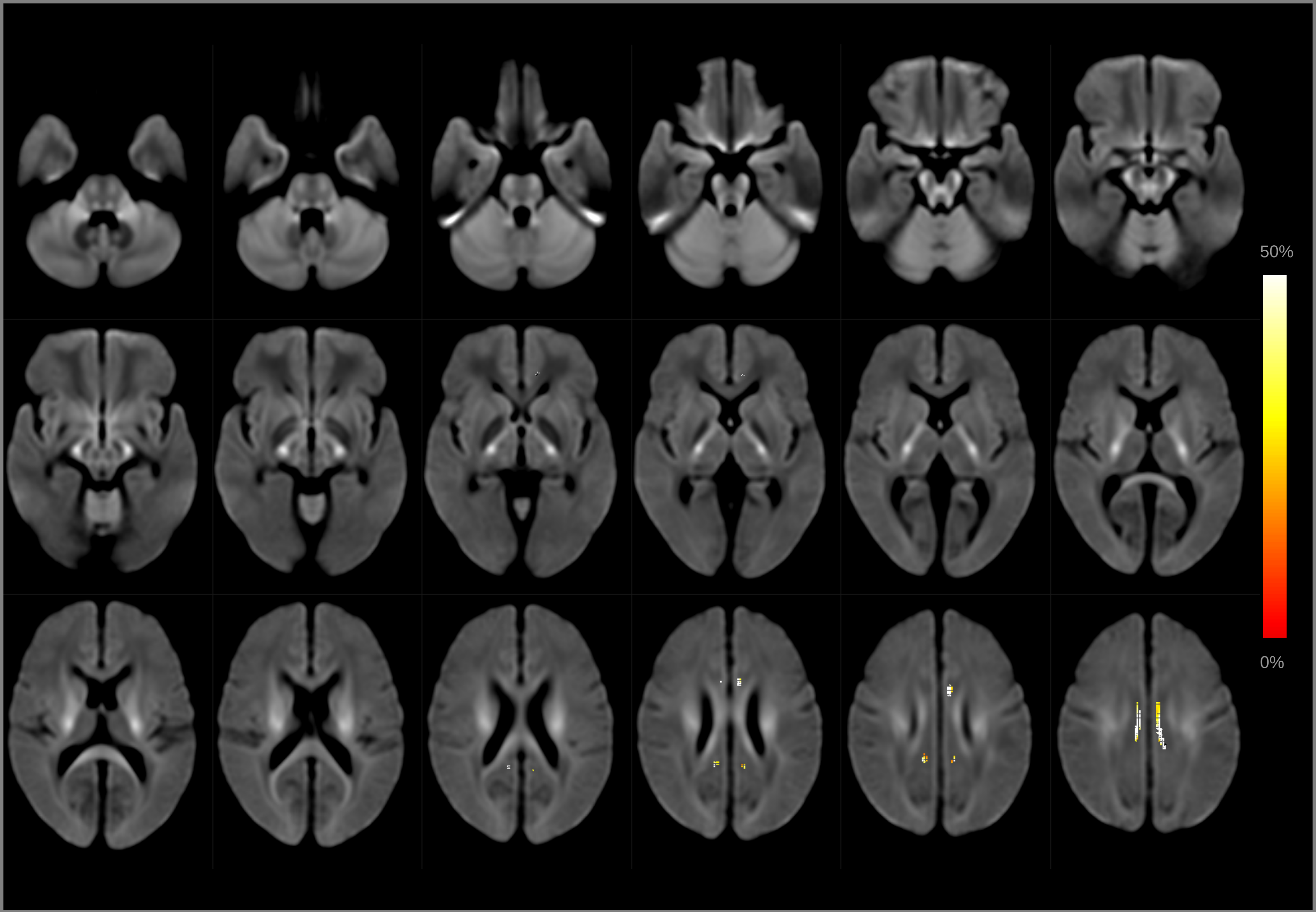

Figure 6: Whole-brain analysis of fixel-wise differences in FDC (Fiber Density and Cross-section) between all preterms and controls, using a general linear model with age, sex and total intracranial volume (TIV) as covariates.

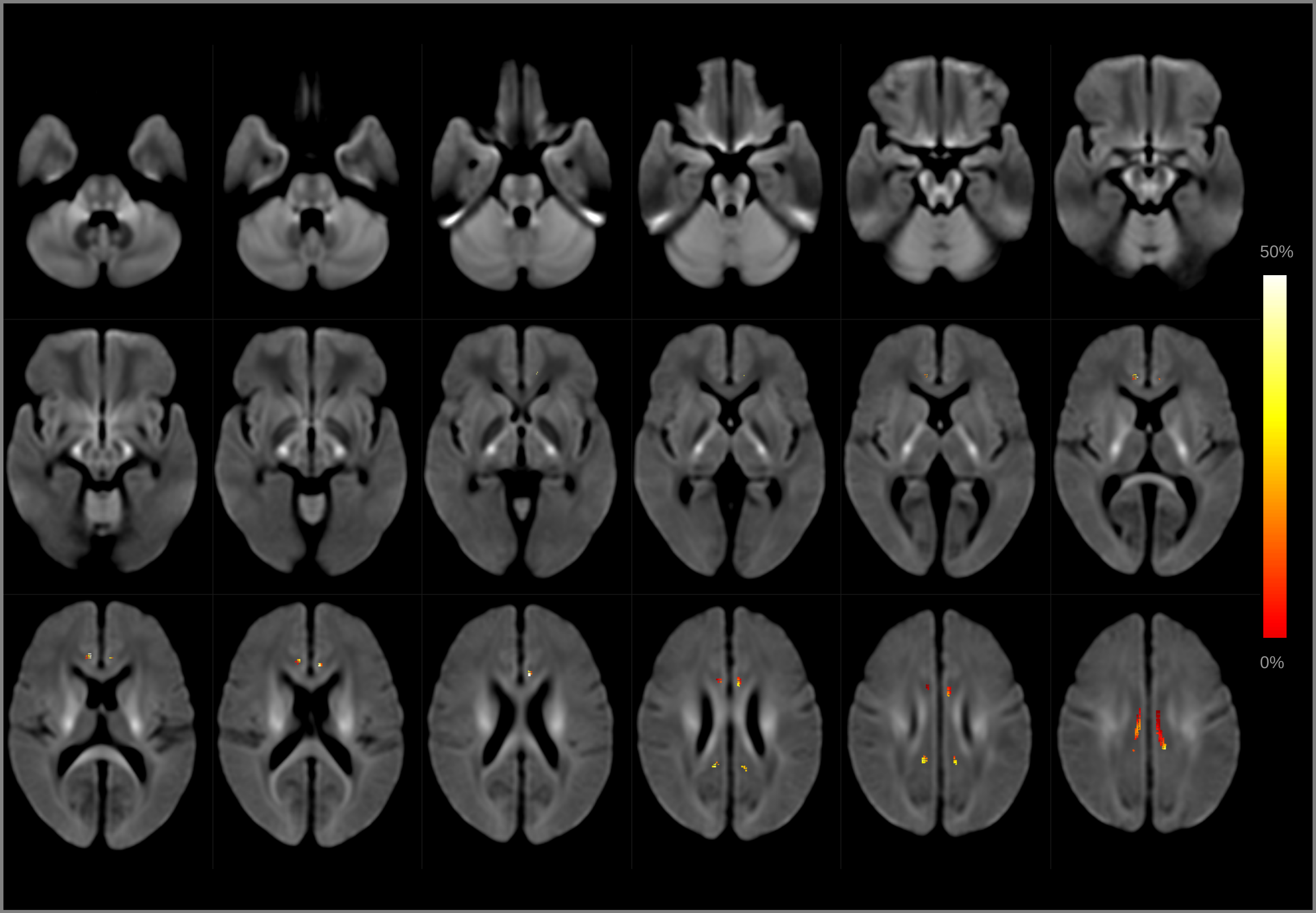

Figure 7: Whole-brain analysis of fixel-wise differences in FD (Fiber Density) between preterms with early postnatal human cytomegaly virus infection and controls, using a general linear model with age and sex as covariates.

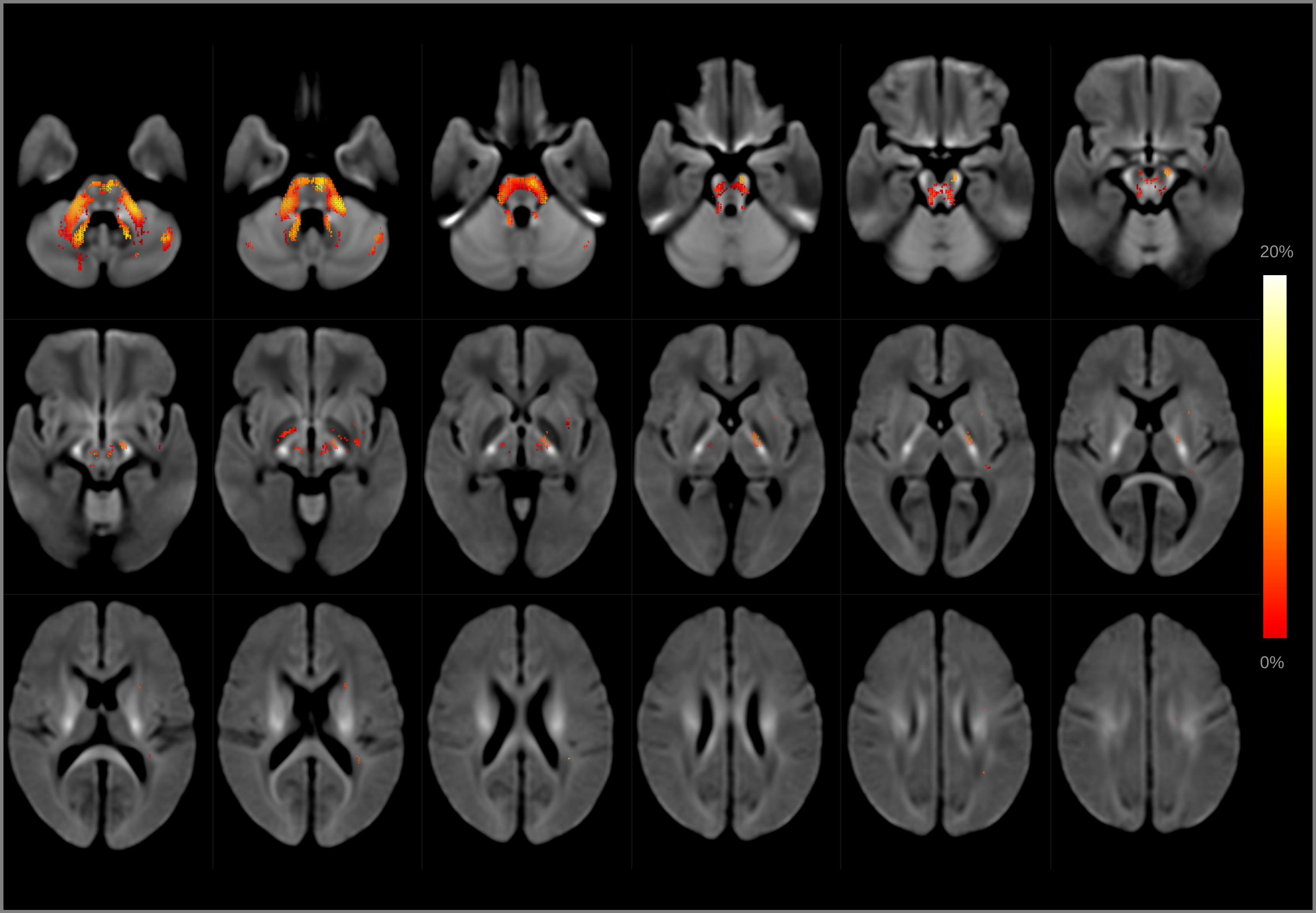

Figure 8: Whole-brain analysis of fixel-wise differences in FC (Fiber Cross-section) between preterms with early postnatal human cytomegaly virus infection and controls, using a general linear model with age and sex as covariates.

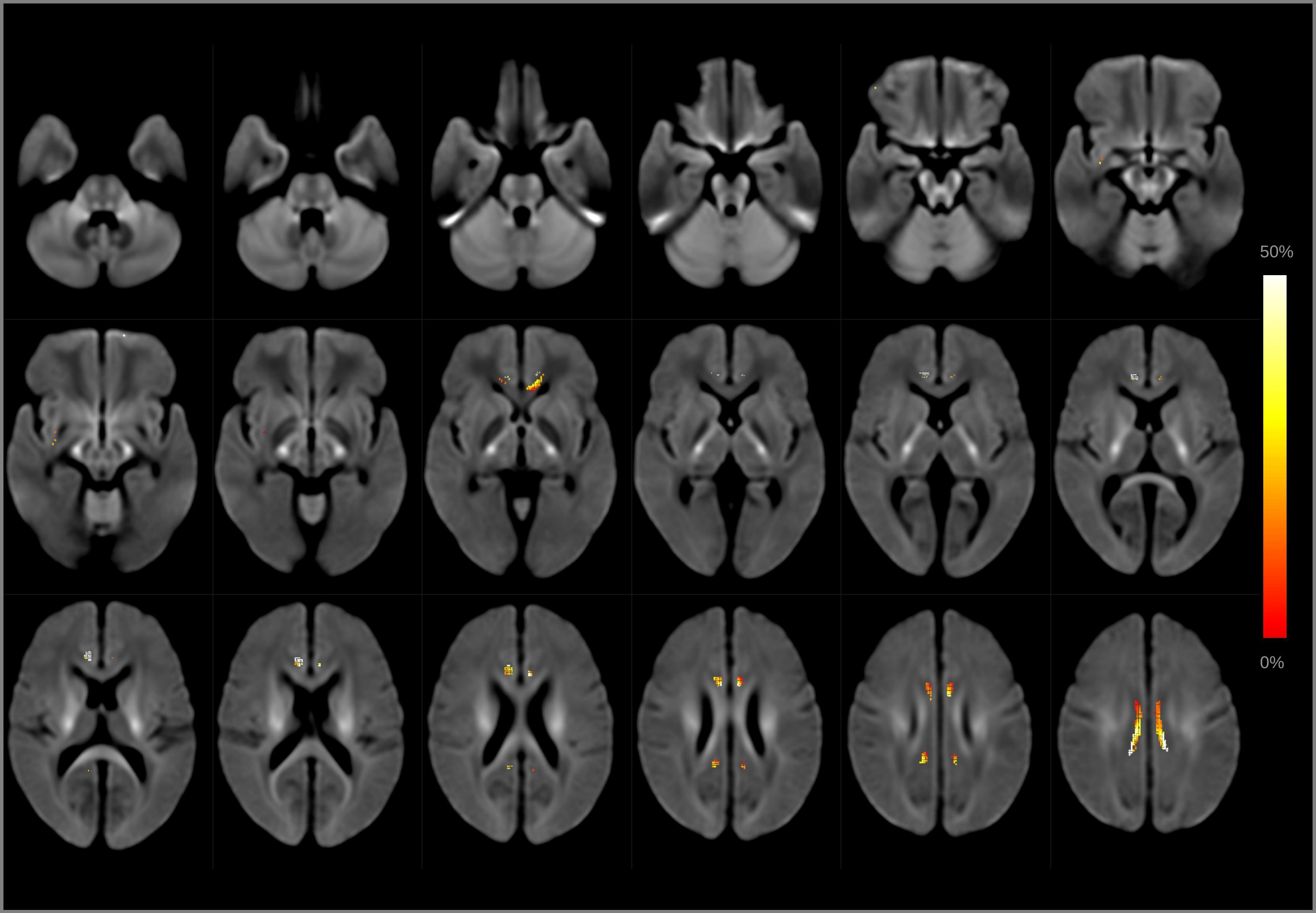

Figure 9: Whole-brain analysis of fixel-wise differences in FDC (Fiber Density and Cross-section) between preterms with early postnatal human cytomegaly virus infection and controls, using a general linear model with age and sex as covariates.

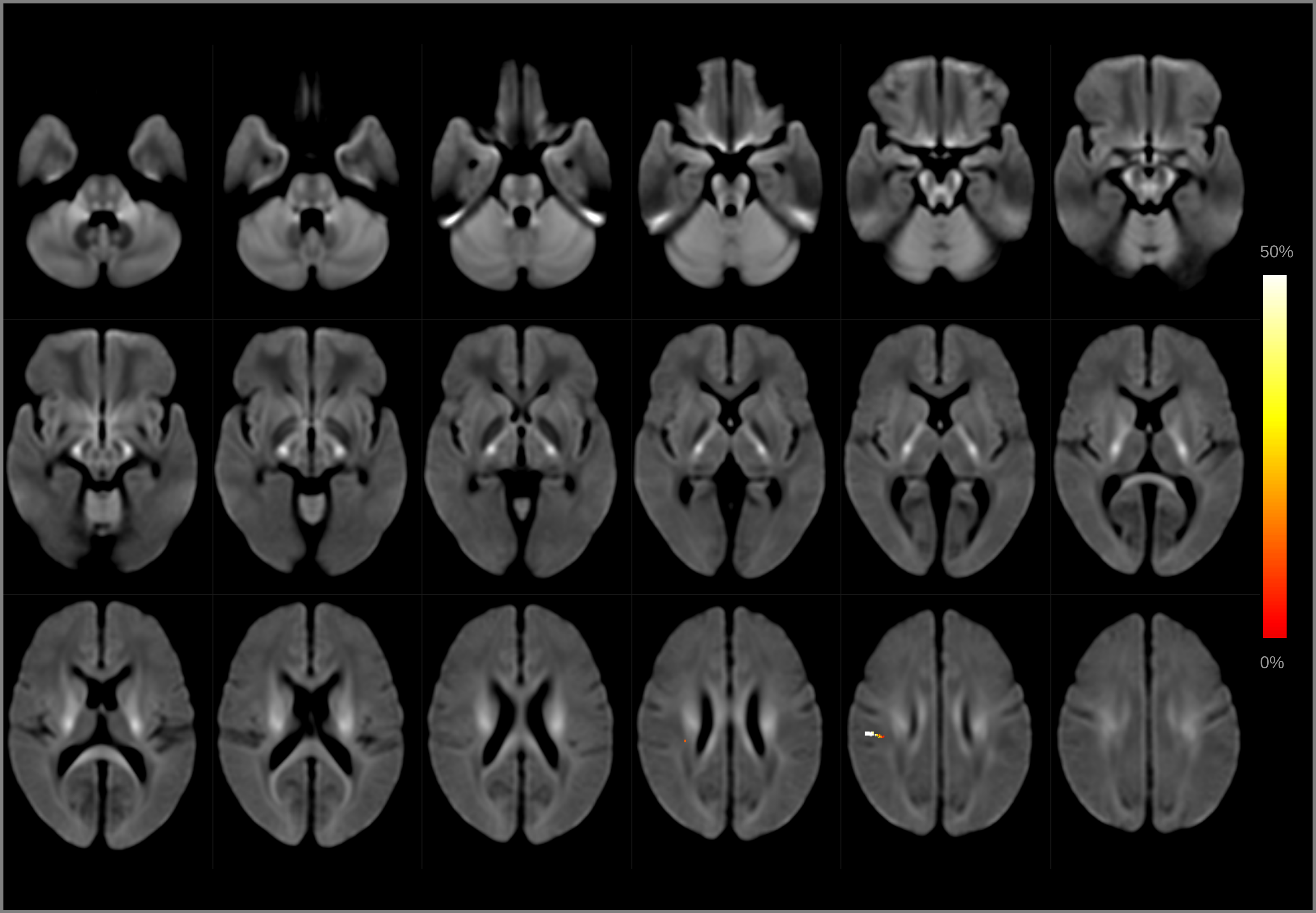

Figure 10: Whole-brain analysis of fixel-wise differences in FD (Fiber Density) between preterms with early postnatal human cytomegaly virus infection and controls, using a general linear model with age, sex and total intracranial volume (TIV) as covariates.

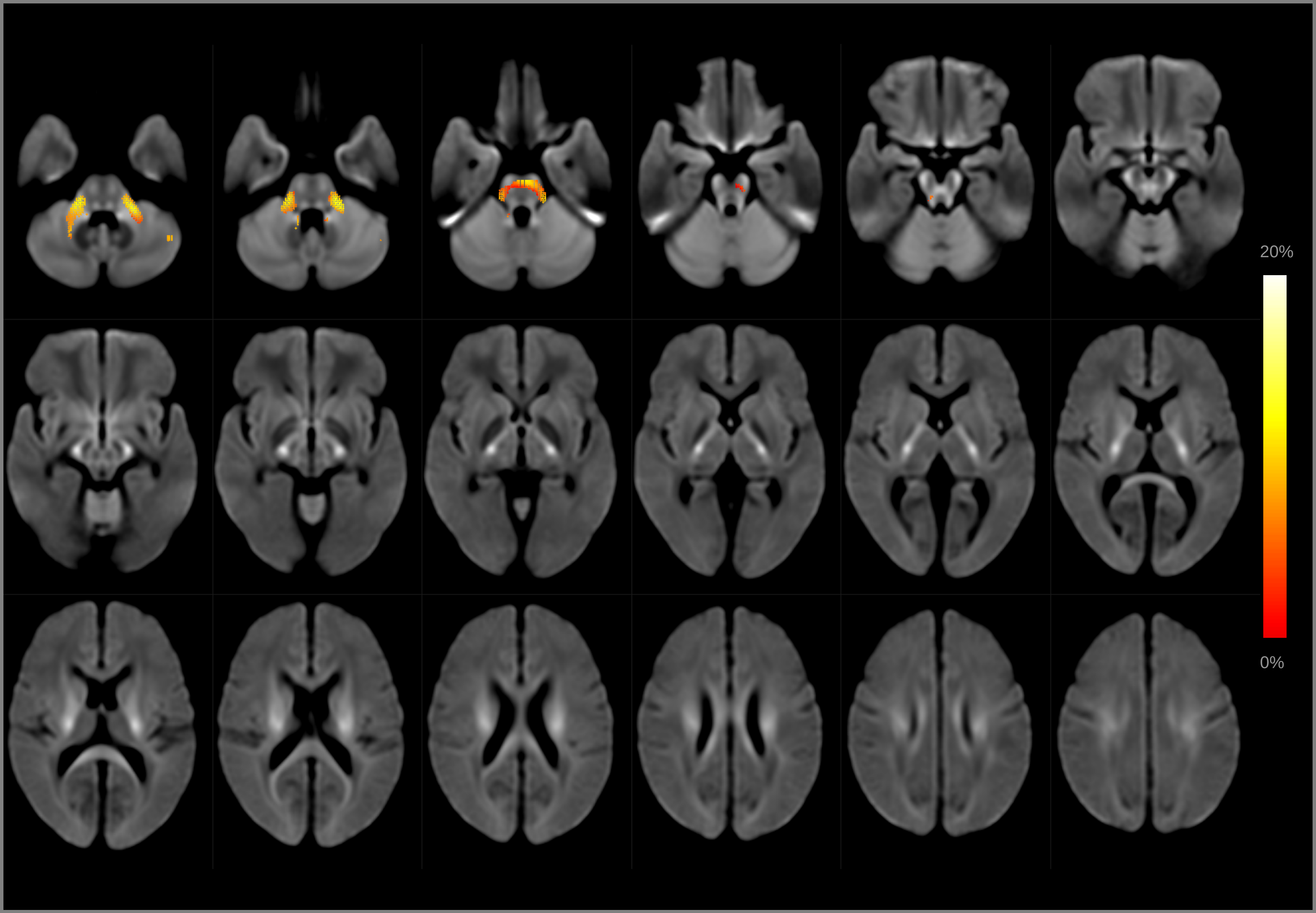

Figure 11: Whole-brain analysis of fixel-wise differences in FC (Fiber Cross-section) between preterms with early postnatal human cytomegaly virus infection and controls, using a general linear model with age, sex and total intracranial volume (TIV) as covariates.

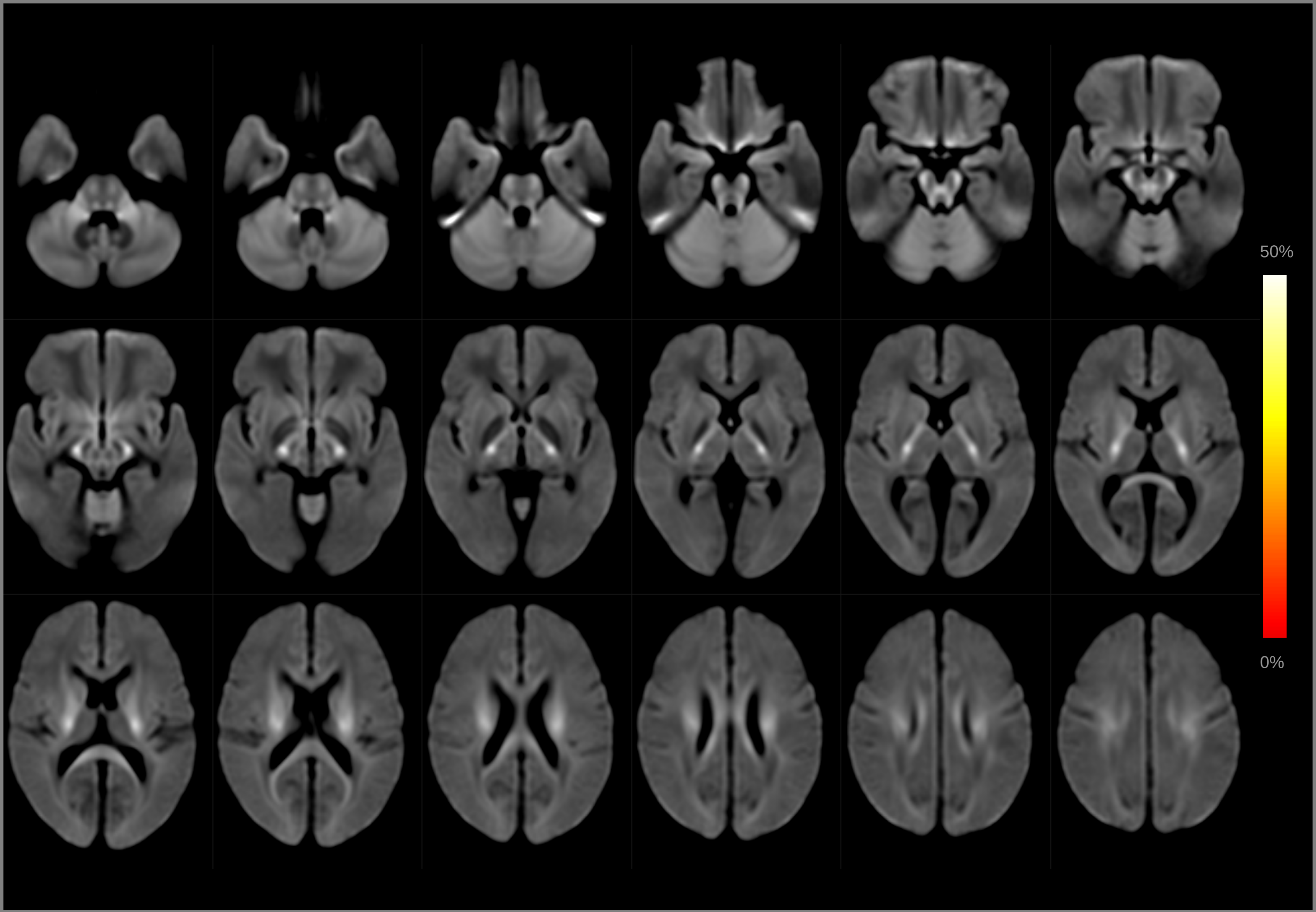

Figure 12: Whole-brain analysis of fixel-wise differences in FDC (Fiber Density and Cross-section) between preterms with early postnatal human cytomegaly virus infection and controls, using a general linear model with age, sex and total intracranial volume (TIV) as covariates.

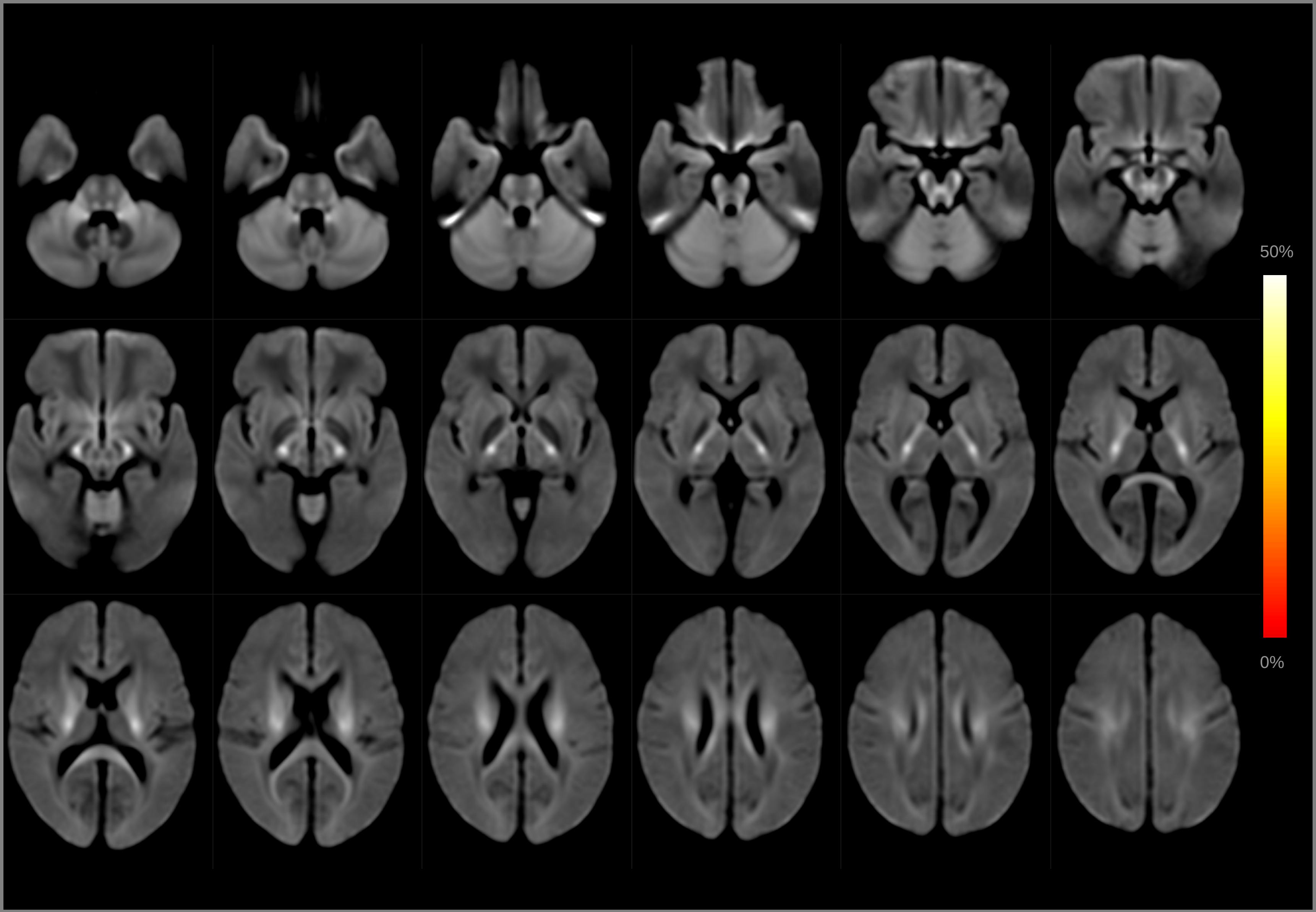

Figure 13: Whole-brain analysis of fixel-wise differences in FD (Fiber Density) between preterms without early postnatal human cytomegaly virus infection and controls, using a general linear model with age and sex as covariates.

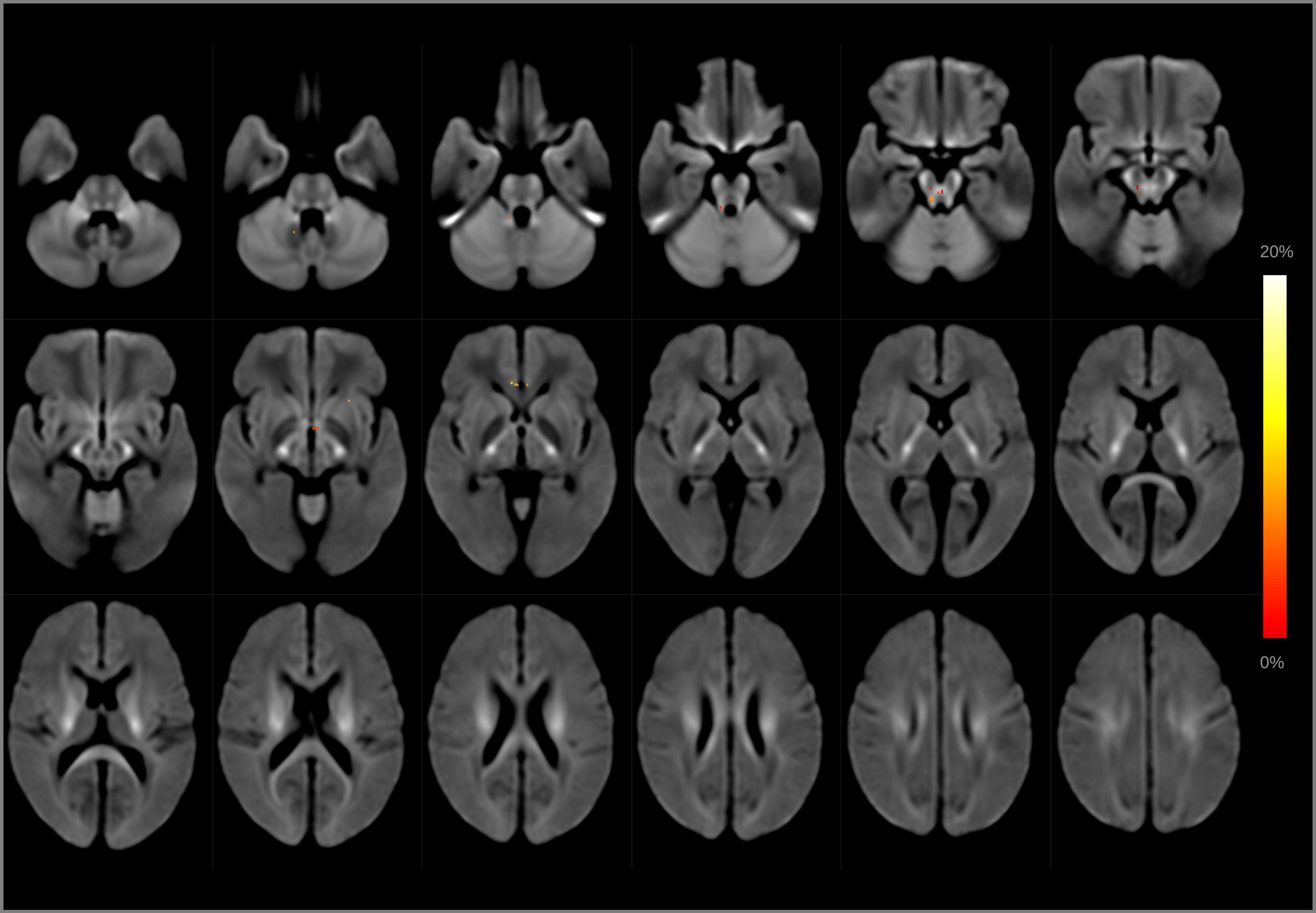

Figure 14: Whole-brain analysis of fixel-wise differences in FC (Fiber Cross-section) between preterms without early postnatal human cytomegaly virus infection and controls, using a general linear model with age and sex as covariates.

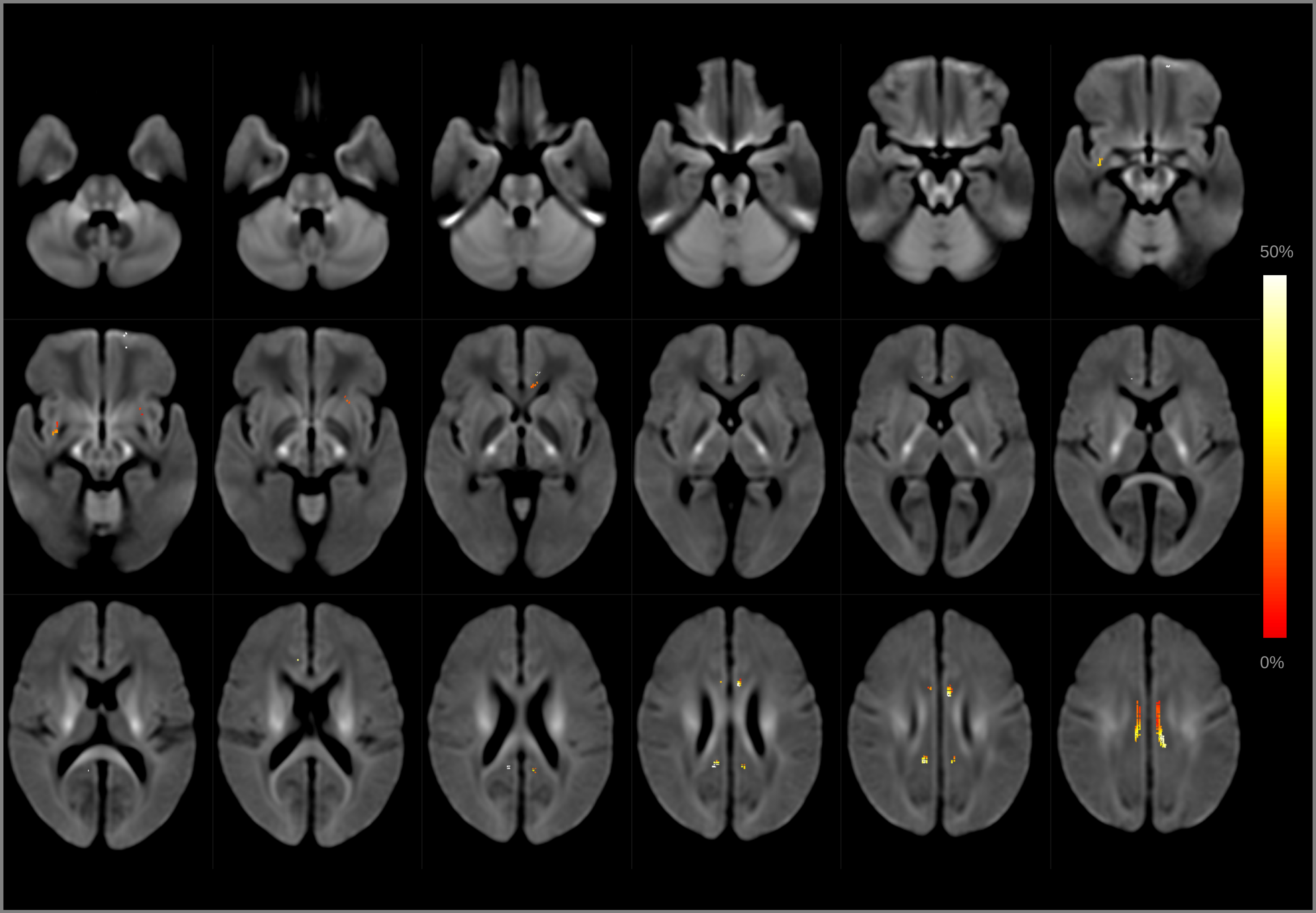

Figure 15: Whole-brain analysis of fixel-wise differences in FDC (Fiber Density and Cross-section) between preterms without early postnatal human cytomegaly virus infection and controls, using a general linear model with age and sex as covariates.

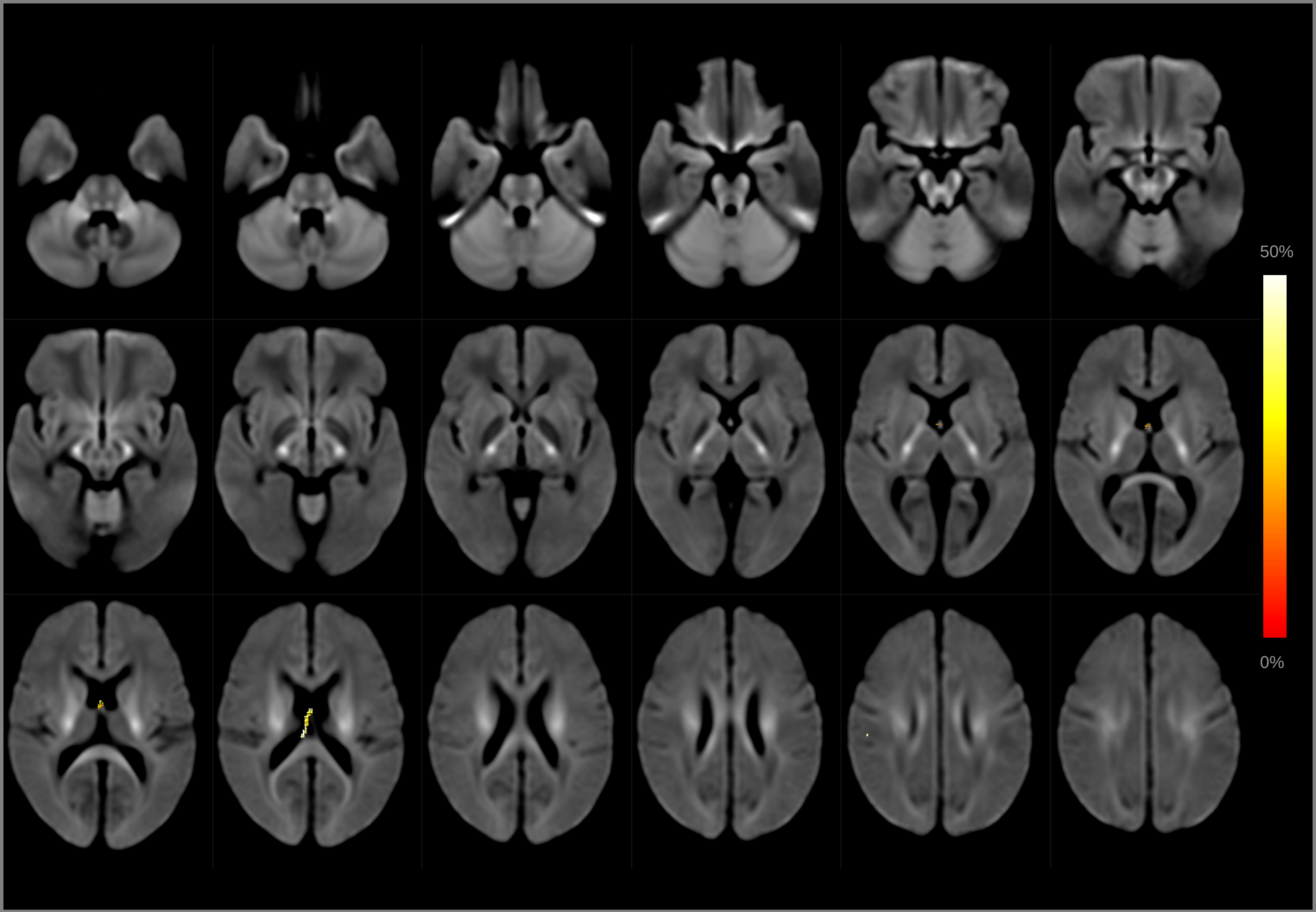

Figure 16: Whole-brain analysis of fixel-wise differences in FD (Fiber Density) between preterms without early postnatal human cytomegaly virus infection and controls, using a general linear model with age, sex and total intracranial volume (TIV) as covariates.

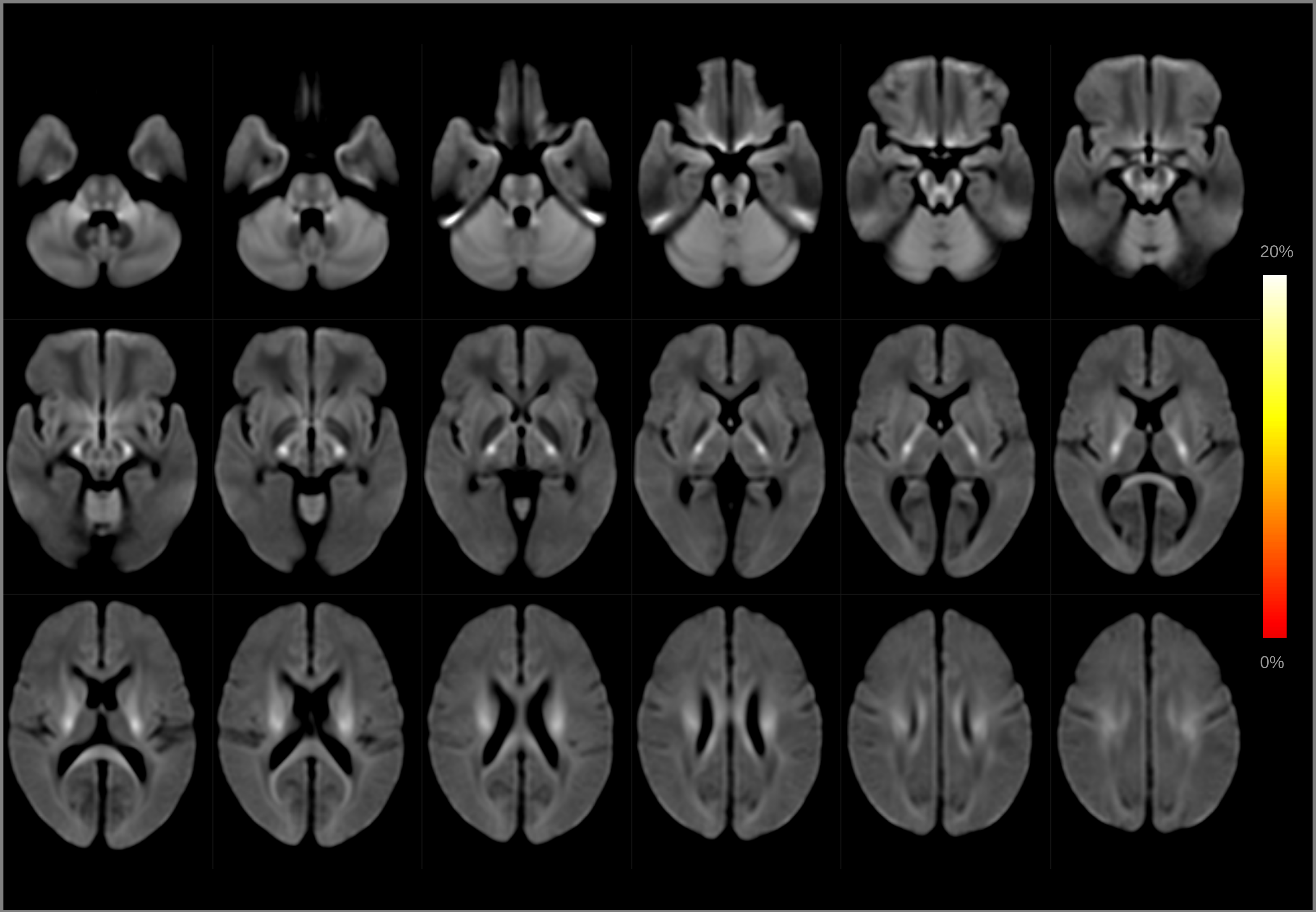

Figure 17: Whole-brain analysis of fixel-wise differences in FC (Fiber Cross-section) between preterms without early postnatal human cytomegaly virus infection and controls, using a general linear model with age, sex and total intracranial volume (TIV) as covariates.

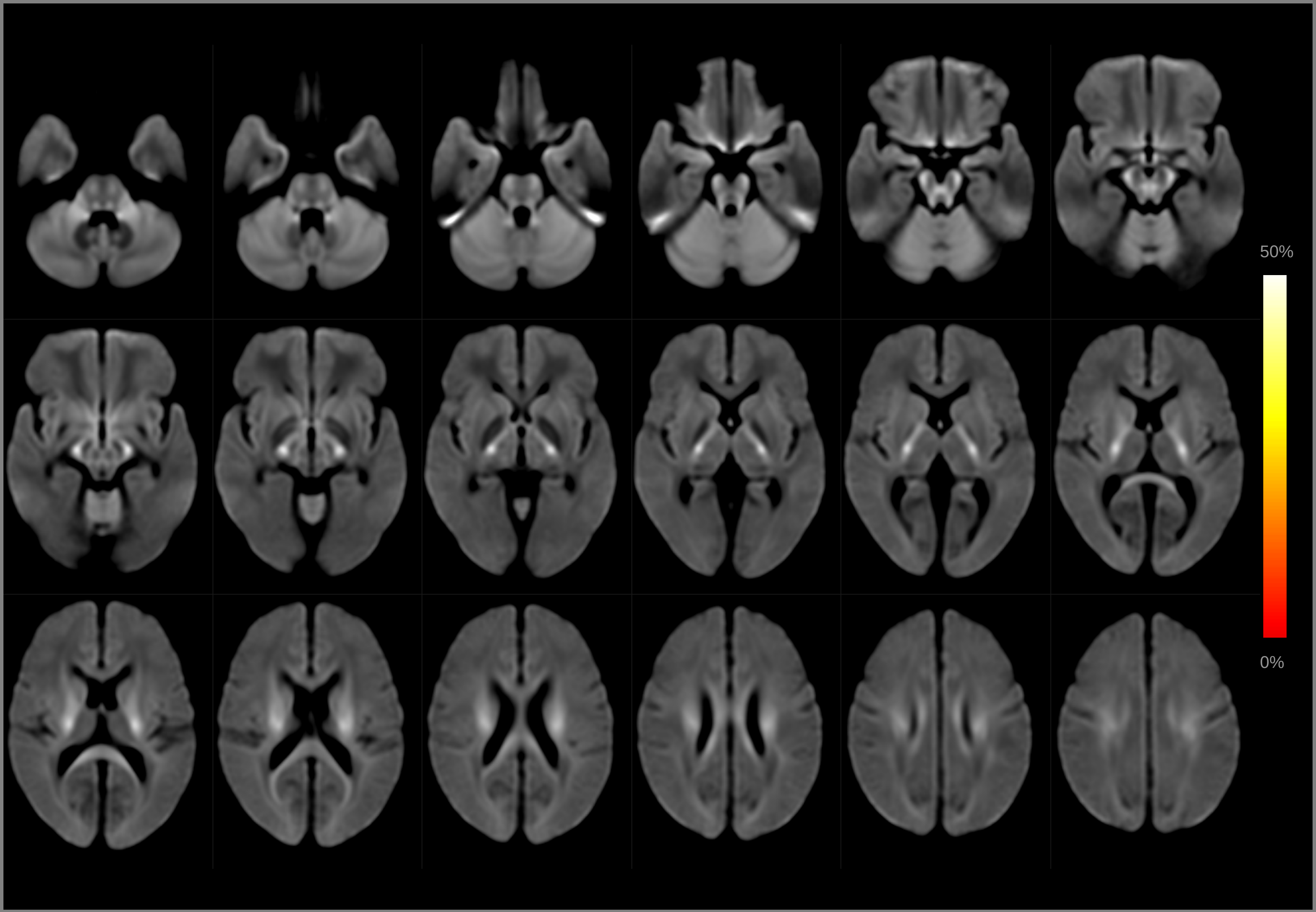

Figure 18: Whole-brain analysis of fixel-wise differences in FDC (Fiber Density and Cross-section) between preterms without early postnatal human cytomegaly virus infection and controls, using a general linear model with age, sex and total intracranial volume (TIV) as covariates.
