# Supplementary material for: Reduced structural connectivity in non-motor networks in children born preterm and the influence of early postnatal human cytomegalovirus infection
Source: Front Neurol. 2023 Oct 2;14:1241387. doi: 10.3389/fneur.2023.1241387 (PMC10577195; doi:10.3389/fneur.2023.1241387)
Supplement: Supplementary file 2 [file Data_Sheet_2.PDF]

Reduced structural connectivity in non-motor networks in children born preterm and the influence of early postnatal hCMV infection

Supplementary Tables - Tract streamline selection criteria

Table 1: Inclusion and exclusion criteria used to identify streamlines belonging to individual tracts. The region numbers refer to the region numbers of the SRI24-Atlas, which was used to locate the respective regions.

Tract	References	Hemisphere	Inclusion regions	Exclusion regions
Fasciculus uncinatus	(Catani et al. 2002) (Kljajevic 2014) (Catani and Thiebaut de Schotten 2008)	left	Supraorbital (5, 9, 15, 25) to temporal pole (83, 87)	Manually drawn between temporal and frontal lobe
		right	Supraorbital (6, 10, 16, 26) to temporal pole (84, 88)	Manually drawn between temporal and frontal lobe
—				
Fasciculus uncinatus	(Catani et al. 2002) (Kljajevic 2014) (Catani and Thiebaut de Schotten 2008)	left	Supraorbital (5, 9, 15, 25) to temporal pole (83, 87)	Manually drawn between temporal and frontal lobe
Cingulum	(Catani et al. 2002) (Catani and Thiebaut de Schotten 2008)	left	31, 33, 35	-
		right	32, 34, 36	-
SLF 1	(Makris et al. 2005) (Kljajevic 2014) (Wang et al. 2016)	left	59 (superior parieteal lobe) and 67 (precuneus) to 1, 3, 7, 11, 13, 19, 23 (entire frontal lobe)	
		right	60 (superior parietal lobe) and 68 (precuneus) to 2, 4, 8, 12, 14, 20, 24 (entire frontal lobe)	
SLF 2	same as SLF 1	left	65 (angular lobe) to 1, 3, 7, 11, 13, 19, 23 (entire frontal lobe)	-

Tract	References	Hemisphere	Inclusion regions	Exclusion regions
		right	66 (Angular lobe) to 2, 4, 8, 12, 14, 20, 24 (entire frontal lobe)	-
SLF 3	same as SLF 1	left	63 (supramarginal lobe) to 1, 3, 7, 11, 13, 19, 23 (entire frontal lobe)	-
		right	64 (supramarginal lobe) to 2, 4, 8, 12, 14, 20, 24 (entire frontal lobe)	-
Fasciculus arcuatus (direct)	(Catani, Jones, and Ffytche 2005) (Kljajevic 2014)	left	81 (superior temporal lobe) to 1, 3, 7, 11, 13, 19, 23 (entire frontal lobe)	
Fasciculus arcuatus (direct)		right	82 (superior temporal lobe) to 2, 4, 8, 12, 14, 20, 24 (entire frontal lobe)	
Fasciculus arcuatus (indirect)		left	65 (angular lobe), 81 (superior temporal lobe), 85 (middle temporal lobe)	
Fasciculus arcuatus (indirect)		right	66 (angular lobe), 82 (superior temporal lobe), 86 (middle temporal lobe)	

Catani, Marco, Robert J. Howard, Sinisa Pajevic, and Derek K. Jones. 2002. "Virtual in Vivo Interactive Dissection of White Matter Fasciculi in the Human Brain." *NeuroImage* 17 (1): 77–94. https://doi.org/10.1006/nimg.2002.1136.

Catani, Marco, Derek K. Jones, and Dominic H. Ffytche. 2005. "Perisylvian Language Networks of the Human Brain." *Annals of Neurology* 57 (1): 8–16. https://doi.org/10.1002/ana.20319.

Catani, Marco, and Michel Thiebaut de Schotten. 2008. "A Diffusion Tensor Imaging Tractography Atlas for Virtual in Vivo Dissections." *Cortex*, Special Issue on "Brain Hodology - Revisiting disconnection approaches to disorders of cognitive function", 44 (8): 1105–32. https://doi.org/10.1016/j.cortex.2008.05.004.

Kljajevic, Vanja. 2014. "White Matter Architecture of the Language Network." *Translational Neuroscience* 5 (4). https://doi.org/10.2478/s13380-014-0232-8.

Makris, Nikos, David N. Kennedy, Sean McInerney, A. Gregory Sorensen, Ruopeng Wang, Verne S. Caviness, and Deepak N. Pandya. 2005. "Segmentation of Subcomponents Within the Superior Longitudinal Fascicle in Humans: A Quantitative, In Vivo, DT-MRI Study." *Cerebral Cortex* 15 (6): 854–69. https://doi.org/10.1093/cercor/bhh186.

Wang, Xuhui, Sudhir Pathak, Lucia Stefaneanu, Fang-Cheng Yeh, Shiting Li, and Juan C. Fernandez-Miranda. 2016. "Subcomponents and Connectivity of the Superior Longitudinal Fasciculus in the Human Brain." *Brain Structure and Function* 221 (4): 2075–92. https://doi.org/10.1007/s00429-015-1028-5.
