# Supplementary material for: Reduced structural connectivity in non-motor networks in children born preterm and the influence of early postnatal human cytomegalovirus infection
Source: Front Neurol. 2023 Oct 2;14:1241387. doi: 10.3389/fneur.2023.1241387 (PMC10577195; doi:10.3389/fneur.2023.1241387)
Supplement: Supplementary file 3 [file Data_Sheet_3.PDF]

Reduced structural connectivity in non-motor networks in children born preterm and the influence of early postnatal hCMV infection

Supplementary Tables - Tractwise analysis results

Table 1: Comparison of tract-averaged fixel metrics between PT and HC, using ANCOVA with age, sex and total intracranial volume (TIV) as covariates for FC and FDC, age and sex as covariates for FD.

**Tractname**	**Side**	**FC**	**FD**	**FDC**
Direct arcuate fascicle	left	n.s.	n.s.	n.s.
Direct arcuate fascicle	right	n.s.	n.s.	n.s.
Indirect Fasciculus arcuatus - posterior part	left	n.s.	n.s.	n.s.
Indirect Fasciculus arcuatus - posterior part	right	n.s.	n.s.	n.s.
Cingulum	left	n.s.	< 0.01	< 0.01
Cingulum	right	n.s.	< 0.01	< 0.01
SLF 1	left	n.s.	n.s.	n.s.
SLF 1	right	n.s.	n.s.	n.s.
SLF 2	left	n.s.	n.s.	n.s.
SLF 2	right	n.s.	< 0.01	n.s.
SLF 3	left	n.s.	0.02	n.s.
SLF 3	right	n.s.	n.s.	n.s.
Uncinate fascicle	left	0.03	n.s.	< 0.01
Uncinate fascicle	right	n.s.	n.s.	0.02

Table 2: Comparison of tract-averaged fixel metrics between CMV+ and HC, using ANCOVA with age, sex and total intracranial volume (TIV) as covariates for FC and FDC, age and sex as covariates for FD.

**Tractname**	**Side**	**FC**	**FD**	**FDC**
Direct arcuate fascicle	left	n.s.	n.s.	n.s.
Direct arcuate fascicle	right	0.03	n.s.	n.s.
Indirect Fasciculus arcuatus - posterior part	left	n.s.	n.s.	n.s.
Indirect Fasciculus arcuatus - posterior part	right	n.s.	n.s.	n.s.
Cingulum	left	n.s.	< 0.01	< 0.01
Cingulum	right	n.s.	< 0.01	< 0.01
SLF 1	left	n.s.	n.s.	n.s.
SLF 1	right	n.s.	n.s.	n.s.
SLF 2	left	0.02	n.s.	0.02
SLF 2	right	n.s.	< 0.01	< 0.01
SLF 3	left	n.s.	n.s.	n.s.
SLF 3	right	n.s.	n.s.	n.s.
Uncinate fascicle	left	n.s.	n.s.	0.01
Uncinate fascicle	right	n.s.	n.s.	0.04

Table 3: Comparison of tract-averaged fixel metrics between CMV- and HC, using ANCOVA with age, sex and total intracranial volume (TIV) as covariates for FC and FDC, age and sex as covariates for FD.

**Tractname**	**Side**	**FC**	**FD**	**FDC**
Direct arcuate fascicle	left	n.s.	n.s.	n.s.
Direct arcuate fascicle	right	n.s.	n.s.	n.s.
Indirect Fasciculus arcuatus - posterior part	left	n.s.	n.s.	n.s.
Indirect Fasciculus arcuatus - posterior part	right	n.s.	n.s.	n.s.
Cingulum	left	n.s.	< 0.01	< 0.01
Cingulum	right	n.s.	< 0.01	< 0.01
SLF 1	left	n.s.	n.s.	n.s.
SLF 1	right	n.s.	n.s.	n.s.
SLF 2	left	n.s.	n.s.	n.s.
SLF 2	right	n.s.	0.04	n.s.
SLF 3	left	n.s.	0.02	n.s.
SLF 3	right	n.s.	n.s.	n.s.
Uncinate fascicle	left	n.s.	n.s.	0.01
Uncinate fascicle	right	n.s.	n.s.	n.s.

Table 4: Comparison of tract-averaged fixel metrics between CMV- and CMV+, using ANCOVA with age, sex and total intracranial volume (TIV) as covariates for FC and FDC, age and sex as covariates for FD.

**Tractname**	**Side**	**FC**	**FD**	**FDC**
Direct arcuate fascicle	left	n.s.	n.s.	n.s.
Direct arcuate fascicle	right	n.s.	n.s.	n.s.
Indirect Fasciculus arcuatus - posterior part	left	n.s.	n.s.	n.s.
Indirect Fasciculus arcuatus - posterior part	right	n.s.	n.s.	n.s.
Cingulum	left	n.s.	n.s.	n.s.
Cingulum	right	n.s.	n.s.	n.s.
SLF 1	left	n.s.	n.s.	n.s.
SLF 1	right	n.s.	n.s.	n.s.
SLF 2	left	n.s.	n.s.	n.s.
SLF 2	right	n.s.	n.s.	n.s.
SLF 3	left	n.s.	n.s.	n.s.
SLF 3	right	n.s.	n.s.	n.s.
Uncinate fascicle	left	n.s.	n.s.	n.s.
Uncinate fascicle	right	n.s.	n.s.	n.s.
