# Supplementary material for: Reduced structural connectivity in non-motor networks in children born preterm and the influence of early postnatal human cytomegalovirus infection
Source: Front Neurol. 2023 Oct 2;14:1241387. doi: 10.3389/fneur.2023.1241387 (PMC10577195; doi:10.3389/fneur.2023.1241387)
Supplement: Supplementary file 4 [file Data_Sheet_4.PDF]

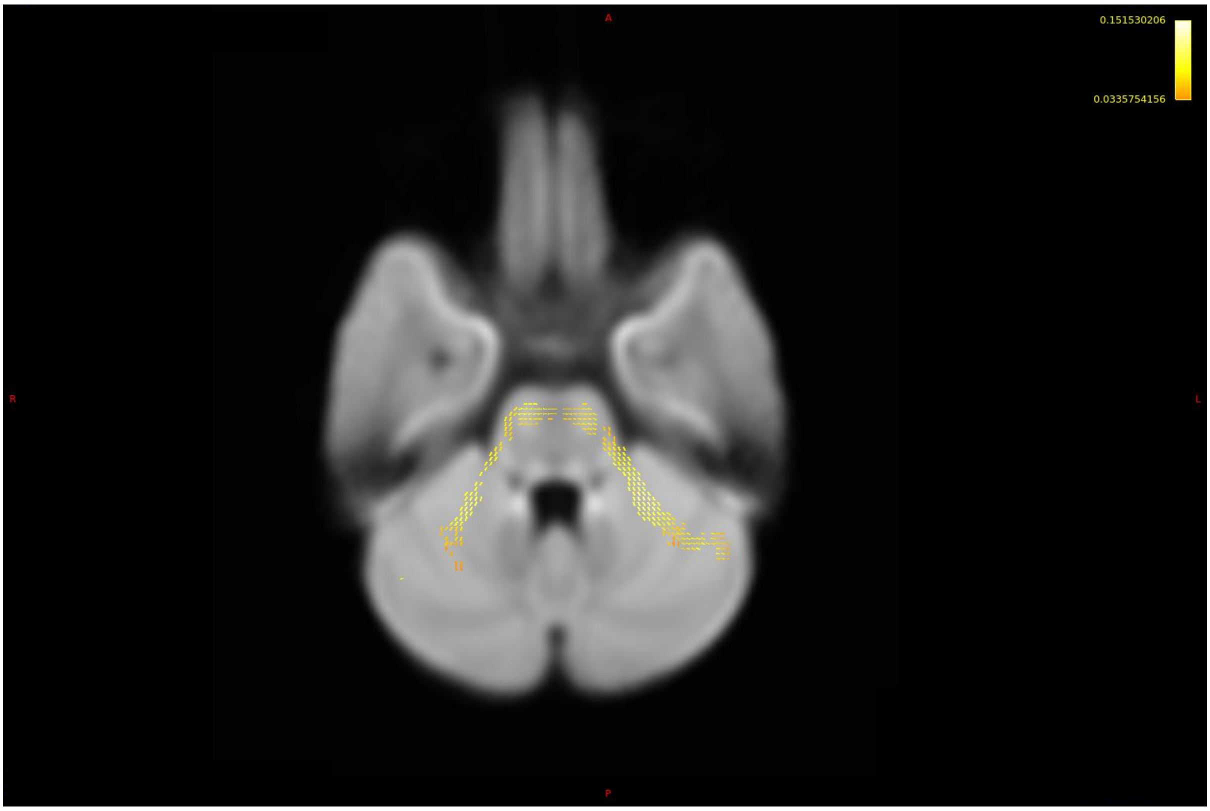

Figure 1: The whole-brain comparison of Fiber Cross-Section (FC) between CMV+ and CMV- infants shows group differences within the cerebellar peduncles and the pontine fibers, after lowering the significance threshold to p=0.2. With p=0.05, no group differences remain significant.

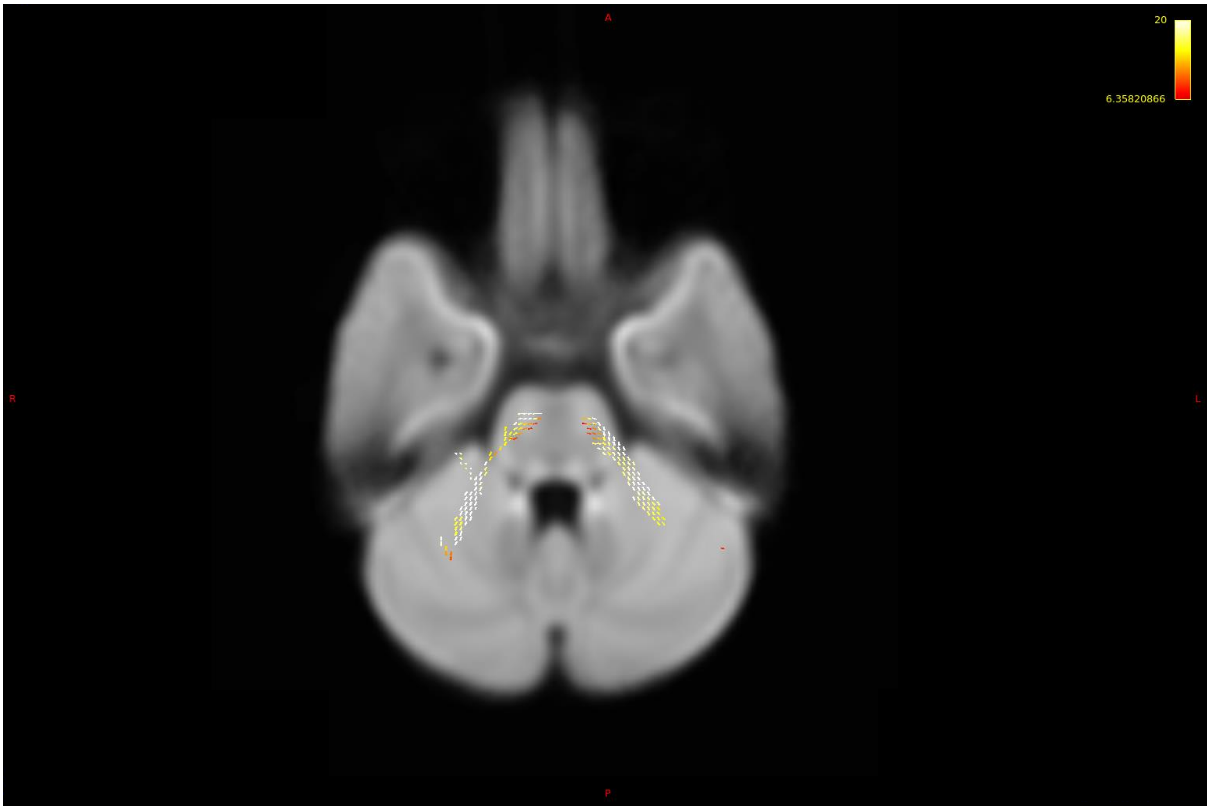

Figure 2: The whole-brain comparison of Fiber Density and Cross-Section (FDC) between CMV+ and CMV- infants also shows group differences within the cerebellar peduncles and the pontine fibers, after lowering the significance threshold to p=0.3. With p=0.05, no group differences remain significant.
